# Topics and trends in artificial intelligence assisted human brain research

**DOI:** 10.1371/journal.pone.0231192

**Published:** 2020-04-06

**Authors:** Xieling Chen, Juan Chen, Gary Cheng, Tao Gong

**Affiliations:** 1 Department of Mathematics and Information Technology, The Education University of Hong Kong, Hong Kong SAR, China; 2 Center for the Study of Applied Psychology, Guangdong Key Laboratory of Mental Health and Cognitive Science and the School of Psychology, South China Normal University, Guangzhou, China; 3 Department of Mathematics and Information Technology, The Education University of Hong Kong, Hong Kong SAR, China; 4 Center for Linguistics and Applied Linguistics, Guangdong University of Foreign Studies, Guangzhou, China; 5 Educational Testing Service, Princeton, NJ, United States of America; Nanjing University of Information Science and Technology, CHINA

## Abstract

Artificial intelligence (AI) assisted human brain research is a dynamic interdisciplinary field with great interest, rich literature, and huge diversity. The diversity in research topics and technologies keeps increasing along with the tremendous growth in application scope of AI-assisted human brain research. A comprehensive understanding of this field is necessary to assess research efficacy, (re)allocate research resources, and conduct collaborations. This paper combines the structural topic modeling (STM) with the bibliometric analysis to automatically identify prominent research topics from the large-scale, unstructured text of AI-assisted human brain research publications in the past decade. Analyses on topical trends, correlations, and clusters reveal distinct developmental trends of these topics, promising research orientations, and diverse topical distributions in influential countries/regions and research institutes. These findings help better understand scientific and technological AI-assisted human brain research, provide insightful guidance for resource (re)allocation, and promote effective international collaborations.

## Introduction

Human brain research aims at achieving a thorough understanding of the structures and functions of human brain. Artificial intelligence (AI) revolutionizes modern human brain research by its tremendous repertoire of technologies and accumulative discoveries while addressing issues about human brain. At the time the mathematician Alan Turing raised the question “Can machines think?” [1], the only recognized systems for conducting complicated computations were biological nervous systems. Therefore, it is common for AI scientists used brain circuits as guidance sources [2]. The multifarious subfields of human brain research provide ample opportunities to validate existing AI methods and develop new ones [3], thus enriching the AI repertoire and enhancing its efficacy in human brain research. Utilizing AI technologies in human brain research has advanced both AI and human brain research and thus made AI-assisted human brain research a fast-growing interdisciplinary field.

Several meta-analysis-based reviews have been conducted on neuroscience-inspired AI and its relevant topics, as summarized in **Table 1**. Back to the seventies and eighties, Arbib [4] synthesized the studies of AI and brain theory and proposed common principles for both fields. Ullman [5] summarized the AI research on brain functions related to visual perception. Recently, Lee and colleagues [6] offered a glimpse on technical principles, clinical applications, and future perspectives of AI technologies in stroke imaging. Hassabis and colleagues [3] revisited the historical interactions between AI and neuroscience, with a stress on shared themes potentially for advancing both AI fields. The majority of existing relevant reviews were conducted by the use of systematic methods. These content-based reviews have two major limitations. First, the efficiency of using manual efforts on content analysis is restricted by the increasing volume of publications, which becomes more explicit due to the proliferation of ‘big data’. Second, research protocols design for conducting coding analyses depends upon the predefinition of conceptual categories. However, such categories are usually not obvious and may change periodically. Third, the numbers of reviewed articles were relatively limited (i.e., from 19 to 350). Besides, the existing reviews focus on narrowed and particular topics, for example, deep learning approaches for glioma imaging [7], machine learning in acute ischemic stroke [8], and AI in stroke imaging [6], failing to provide a general overview of the community of AI-enhanced human brain research. In addition, these qualitative reviews on specific topics or bibliometric analyses based primarily on metadata of scientific publications (e.g., year of publication or citation index) cannot accommodate the wide and fast-growing research and application scopes of modern AI-assisted human brain research.

**Table 1:** Recent reviews on AI-enhanced neuroscience research and its relevant topics.

Reviewer(s) and year	Research topic	No. of articles	Method	Period	Analysis aspects
Xu et al. (2019) [9]	Magnetic resonance imaging and AI for Parkinson’s disease diagnosis	71	Systematic review	1990–2019	To review studies in three subfields: diagnosis, differential diagnosis, and subtyping of Parkinson’s disease, to depict the general workflow from magnetic resonance image to classification results, and to summarize an essential assessment of the recent research and to offer suggestions for future research.
Shaver et al. (2019) [7]	Deep learning approaches for glioma imaging	12	Systematic review	2009–2018	To summarize recent applications of deep learning to detect glioma and predict outcome, with foci on pre- and post-operative tumor segmentation, genetic characterization of tissue, and prognostication.
Sakai, K and Yamada (2019) [10]	Machine learning studies on major brain diseases	209	Systematic review	2014–2018	To summarize detailed information such as machine learning approaches, sample size, inputted features types and reported accuracy.
Kamal et al. (2018) [8]	Machine learning in acute ischemic stroke neuroimaging	10	Systematic review	2011–2018	To summarize detailed information such as machine learning approaches, features, and results.
Senders et al. (2018) [11]	Machine learning for predicting neurosurgical outcome	30	Systematic review	1998–2017	To offer an overview of the theoretical concepts of machine learning and to examine its usefulness to assist neurosurgical decision making, and to compare the performance of machine learning with prognostic indices, traditional statistical approaches, and clinical experts.
Lee et al. (2017) [6]	AI in stroke imaging	49	Systematic review	till 2017	To provide an overview of the applications of AI in stroke imaging, with particular foci on technical principles, clinical applications, and future perspectives.
Sotoudeh et al. (2019)	AI in the management of glioma	84	Systematic review	till 2019	To offer a succinct depiction of the foundational concepts of AI techniques and their relevance to clinical medicine, and to review innovative AI techniques in glioma diagnosis and management.
Sotoudeh et al. (2019) [12]	AI for mental health and mental illnesses	28	Systematic review	2015–2019	To review AI’s applications in healthcare, to discuss how AI could facilitate clinical practice, issues requiring further study, and ethical implications concerning AI technologies.
Aneja et al. (2019) [13]	Artificial intelligence in neuro-oncology	27	Systematic review	2017–2019	To discuss current adoption of AI within neuro-oncology and to demonstrate emerged challenges in the integration of AI in clinical practice.
Senders et al. (2018) [14]	Machine learning in neurosurgical care	221	Systematic review	till 2017	To summarize detailed information such as treatment stages, disease conditions machine learning methods inputted features neurosurgical applications, and results.
Hassabis et al. (2017) [3]	Neuroscience-inspired AI	187	Systematic review	till 2017	To review interactions between AI and neuroscience and to demonstrate latest progresses in AI motivated by research of neural computations.
Chen et al. (2019) [15]	Human brain study using AI	6317	bibliometric analysis	2009–2018	To analyze distributions of annual article and citation counts, identify productive journals and institutions, visualize scientific collaborations, and to uncover the most frequently used keywords.

This study is built on the study by Chen et al. (2019) [15], which focused on the analyses of the distributions of annual article and citation counts, research subject distribution, productive journals and institutions, scientific collaborations, as well as the frequently used keywords. Although they use the same dataset as this study, the research foci and the research methods adopted are totally different. Specifically, this study centres on detection of research topics covered within the AI-assisted human brain research articles, particularly with the use of an innovative text mining method, namely structural topic modeling (STM). In face of the increasing diversity of research topics and technologies in this field, there is a necessity of quantitative studies that help better understand the following issues:

1. *What are the prominent research topics in this interdisciplinary field*?
2. *How do these research topics evolve with time*?
3. *What are the distributions of these topics across different types of research units*?

Answers to these questions can provide a comprehensive depiction and a state-of-the-art understanding of AI-assisted human brain research, as well as useful suggestion for its future development.

To address these issues, this study combined the STM with the bibliometric analysis to conduct a quantitative investigation on the scientific publications of AI-assisted human brain research in the past decade. We first created the dataset for analysis by extracting the research papers from the Science Citation Index Extended (SCIE) and Social Science Citation Index (SSCI) databases provided by the ISI Web of Science using the modified expert-designed queries (see section 2 Material and methods for how to construct these queries, and **S1** and **S2 Tables** for the complete keywords lists in AI and human brain research used for data retrieval). After data filtering following the designed criteria (see **Table 2**), we applied STM to identify prominent topics from the remaining papers in the dataset, and the Mann-Kendall (MK) trend test to capture the temporal shifts in topical prevalence over the past decade. In addition, we conducted correlation and cluster analyses to visualize the relations between identified topics. Furthermore, we compared the topical distributions among the top 20 influential countries/regions and institutes to reveal the contributions of different research units. Based on the collaboration networks of those countries/regions and typical centrality measures, we discussed the importance and collaborative patterns of different countries/regions. These findings could lead to insightful implications guiding researchers and project investigators in this field.

**Table 2:** Inclusion and exclusion criteria for manual verification of the retrieved papers.

Area	Type	ID	Criteria
Human brain research	Inclusion criteria	I1	Human brain anatomy
		I2	Human brain functions
		I3	Human brain diseases
		I4	Treatments for human brain diseases
		I5	Methods for brain signal collection or analysis
	Exclusion criteria	E1	Not focused on human
		E2	Not focused on brain
		E3	Not a scientific research
		E4	Without abstract
AI research	Inclusion criteria	I1	Use of AI algorithms/approaches/technologies
		I2	Improvement of AI technology/algorithm
	Exclusion criteria	E1	Use of pure mathematical or statistical algorithms
		E2	Use of automatic methods rather than AI methods
		E3	Use of computer algorithms rather than AI algorithms
		E4	Without abstract

Although the findings in this paper are limited to AI-assisted human brain research, the combinatorial approach and the analytic framework proposed are domain-independent and have several significances. On the one hand, combining STM with bibliometric analysis bears many benefits. For example, it makes bibliometric analysis adaptive to large-scale textual data beyond scientific publications. In addition, it can reflect the practical issues in the whole life cycle of research development, since the data are obtained using scientific methods [16]. Integrating the cutting-edge text mining approaches with the time-honoured bibliometric approach forms a robust empirical framework which situates fine-grained discursive results in the large textual data sources.

On the other hand, systematic analyses on the developmental trends, correlations, and clusters of prominent topics, as well as the interactions between these topics can explicitly answer more straightforward questions such as what are happening in a research field and what will happen in future, thus helping shape research priorities. Knowledge of how research priorities gradually emerge is important when it comes to understanding the role that science plays in society. In addition, identifying substantial topics, their proportions and trends, and emerging research areas around those topics, especially in a way of longitudinal and sustained monitoring, can efficiently capture the core of a research field, track its present and future developments, and address concerns about resource (re)allocation among diverse disciplines and research areas. These support and benefit scientific research, management of technology and innovation, and entrepreneurship in general.

## Material and methods

**Fig 1:**
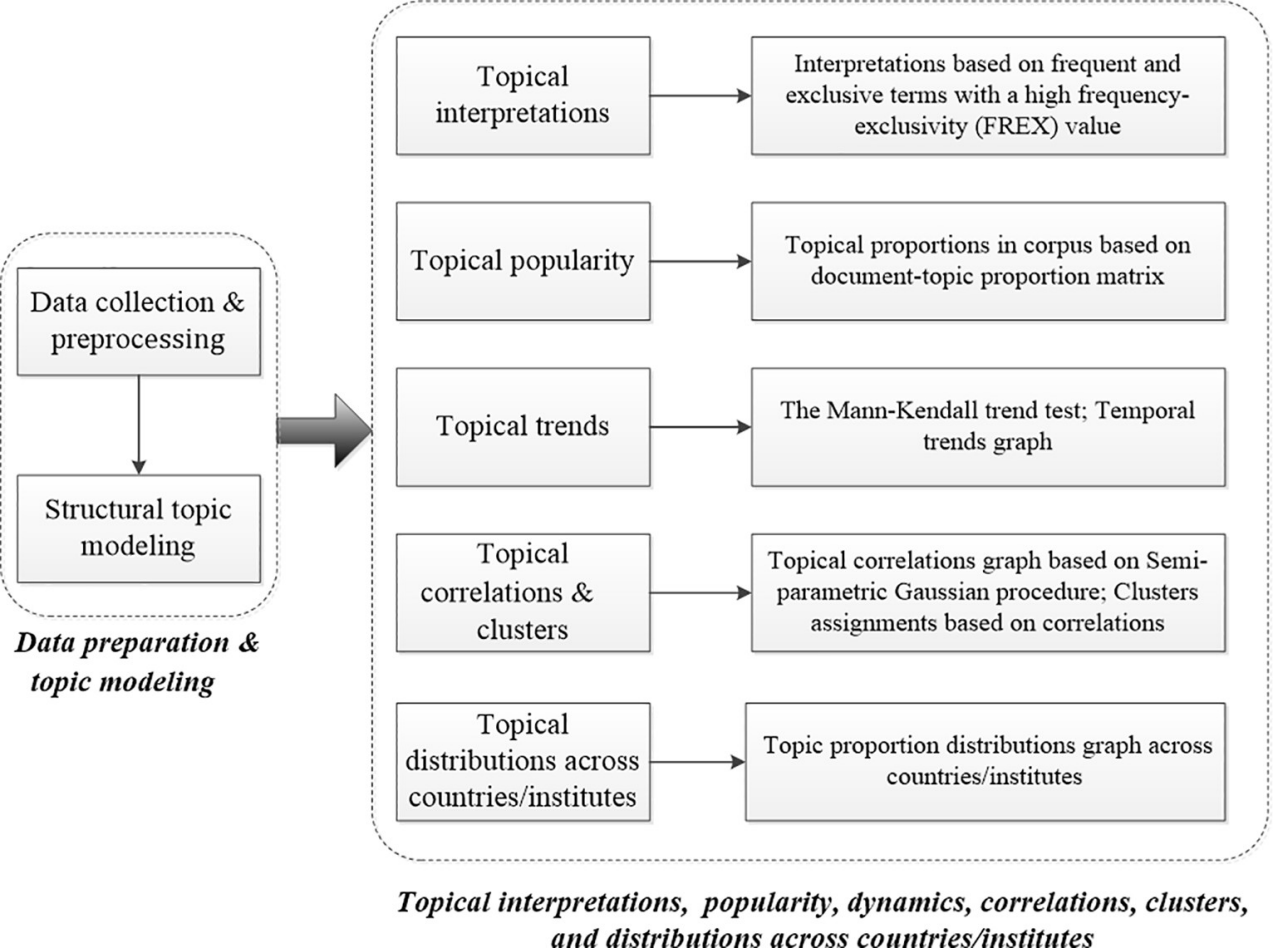
Proposed analytic framework.

The STM-based bibliometric analytic framework proposed in this study is shown in **Fig 1**. It consists of data preparation and pre-processing, as well as topical interpretations, popularity, dynamics, correlations, clusters, and distributions across countries or institutes.

### Data preparation

The data for analysis was retrieved from the SCIE and SSCI databases on Web of Science (www.webofknowledge.com). As strictly selected academic databases, SCIE and SSCI are well-known academic literature indexing tools with documents published on peer-reviewed and high-quality journals [17], and have been widely used in bibliometric or scientometric studies.

A critical procedure during data retrieval was to design keyword queries for AI and human brain research, respectively, and then use such queries to retrieve literature from the bibliography databases. A challenge here was to maximize the identification of the studies concerning AI and human brain. For example, papers specific to AI may not mention terms like ‘artificial intelligence’, thus a query to retrieve all papers in AI research must contain field-specific terms, such as ‘machine intelligence’.

In line with [18], we took the following steps to obtain the keyword queries. As for the keyword query for AI, domain experts first provided a list of seed keywords concerning AI. Some examples of such keywords were ‘machine learning’, ‘natural language processing’, or ‘image recognition’. We then used this query of seed keywords to retrieve the papers containing such keywords in titles, abstracts, or author defined keywords. After that, we collected all the author defined keywords from the highly-cited papers (according to the Essential Science Indicators, which had been cited enough as of January/February 2019 to be placed among the top one percentage of their academic fields) retrieved. These collected keywords were presented again to the domain experts, who might exclude some irrelevant words. Then, the relevant keywords left were added to form the final keywords query for AI.

Similarly, the keywords query for human brain research was also obtained. Two kinds of seed keywords related to human brain research were considered here. The first kind included the keywords definitely related to human brain research, such as ‘brainnetome’, ‘brain mapping’, ‘electroencephalogram’, and ‘functional magnetic resonance imaging’. The second kind included the keywords that might co-occur with some ‘brain’ qualifiers (e.g., ‘functional magnetic resonance imaging’, or ‘brain’), such as ‘emotion’ and ‘memory’.

Using the final keyword queries, we accessed to the SCI and SSCI databases on March 27, 2019 to collect the target papers. There were three searching criteria:

1. The papers must be written in English and published during the years 2009–2018;
2. The type of the papers must be ‘article’, on account that they usually provide more original research findings and contain explicit information about authors and their institutes [19];
3. The terms in the title, abstract, or keywords of each paper must match at least one of the keywords in the final queries.

Based on these criteria, we obtained 30,316 papers with full bibliographic information of annual citations. Key elements of each paper, such as title and author(s) address(es), were extracted using an in-house Python program. Duplicated data were deleted according to the information of title, journal, year of publication, and author.

Data filtering was conducted to ensure not only a close alignment of the data to the research goal, but also the efficiency and reliability of the analysis. Considering that the abstract of a paper usually specifies its research object, key problems and results, following [20], we included the abstracts of the collected papers as the primary materials for text mining. Thus, papers without abstracts (usually book chapters or short reports) were excluded. Then, domain experts carried out the filtering [21] separately based on the criteria provided in **Table 2**. For instance, from an AI perspective, one domain expert reviewed all the papers according to the criteria. Another domain expert performed the review process based on the same criteria, but only for 1,000 randomly selected papers from the whole retrieved dataset. The consistency rate between the two experts was around 95%, indicating that the filtering results were reliable and acceptable. A similar process was applied to the review of the human brain papers by another two relevant domain experts (the consistency rate was above 90%).

In total, 6,317 papers were selected to form the final dataset for analysis. The bibliographic information of each paper was confirmed and recorded according to the original articles. The names of the authors, institutes, and countries/regions were further extracted from the address information and confirmed and reviewed manually to ensure consistent expressions. Papers from Hong Kong, Macau, and Taiwan were calculated separately, while papers from England, Scotland, Northern Ireland, and Wales were unified as from UK.

### Structural topic modeling

Topic models are text mining techniques for extracting hidden thematic structures within large scale documents [22]. Various types of topic models have been proposed and adopted in various domains (e.g., [23–26]). Structural Topic Modeling (STM) [27, 28] is a newly developed topic model to assess substantial textual data and extract semantic information using statistical algorithms. In this study, we used STM to uncover latent topics in the papers of AI assisted human brain research. In STM, each paper is assumed as a mixture of multiple, correlated topics, each with characteristic terms along with its own prior distribution. Estimation of the latent topics is conducted in a way that regards each paper as a mixture of correlated topics, and meanwhile, incorporates paper-level external covariates into the prior distributions of paper topics or topic words [29].

The modeling process was conducted using the R package *stm* [27]. To guarantee high analysis efficiency, pre-processing of the analysis units, i.e., title, abstract, author defined keywords, as well as KeyWords Plus (index terms automatically generated from the titles of cited papers provided by Web of Science) data, was needed before modeling. First, all collected terms were converted to lower case. Second, numbers, punctuations, and common stop terms like ‘an’, ‘a’, and ‘in’, as well as terms with broad meanings such as ‘paper’, ‘method’, and ‘analyze’, were removed, as they appear in almost every paper. Third, as indicated in [30], the importance of different parts of a paper varies, so do the terms from those parts. Accordingly, we assigned the weights to the terms from the keywords, titles, and abstracts as 0.4, 0.4, and 0.2 separately.

Since STM is an unsupervised method, one needs to decide how many topics are estimated. We followed the decision-making process proposed in [31], which requires considerable qualitative discernment by domain experts having deep understanding of the dataset. In this study, we fitted candidate models with 10, 20, 25, 30, 35, and 50 topics. The domain experts recursively assessed the interpretability and relative efficacy of each model according to their expertise as well as substantive knowledge of the issue at hand. In this way, we selected a 30-topic model having the highest external validity and the most semantically coherent output of distinctive topics without impeding topic interpretability.

### Mann-Kendall trend test

After modeling, we counted the proportion of each topic as a representation of their popularity in the research field, as in Eq (1), where *P*_*k*_ denoted the proportion of the *k*_*th*_ topic, *θ*_*d*,*k*_ was the proportion of the *k*_*th*_ topic in the *d*_*th*_ paper, and *D* was 6317.

$$P_{k}=\frac{\sum_{d}\theta_{d,k}}{D}$$ (1)

We then counted the proportion of the *k*_*th*_ topic in year *t* using Eq (2) for the temporal trend analysis. Here, *py*_*d*_ represented the publication year of the *d*_*th*_ paper, and *D*_*t*_ was the number of papers in year *t*.

$$P_{k,t}=\frac{\sum_{d|py_{d}=t}\theta_{d,k}}{D_{t}}$$ (2)

We employed the non-parametric Mann-Kendall test [32] to examine annual trends of the identified 30 topics.

### Bibliometrics and indicators

Due to rapid development of computers, bibliometric analysis has received more attention recently and been increasingly accredited as an important tool of using objective criteria to measure scholarly quality and productivity in a specific research area [33]. It not only boosts the historical research retrospectives but also helps explore objectively the research hotspots and frontiers in specific disciplines from both macro and micro perspectives, thus serving as useful supplement to the views of domain specialists [34]. Bibliometric analysis has been employed in various disciplines to describes distributive patterns of literature on a particular field [35–43].

Performance analysis is one of the main methods in bibliometrics. Because of computation easiness and capability in balancing quantity and quality, *h*-index and its variants have played significant roles in academia [44]. The *h*-index combines the number of papers and their impact, thus simplifying the characterization of a researcher’s scientific outputs [45]. It has been extended to measure the scientific impact of a country/region, an institute, and a journal.

In addition to the *h*-index, we also considered two other popular bibliometric indices, namely, paper and citation counts, which measure productivity and influence, respectively. The total numbers of papers of countries/regions, institutes, as well as journals focus on different types of scientific actors. The number of citations of a research paper reflects its scientific community [46]. Citation count was also used to evaluate scientific impact of countries/regions, institutes, and journals.

## Results

### Topic identification

The dataset for analysis consists of 6,317 AI-assisted human brain research papers, which contain 532,373 single words (5,418,800 characters). Among these words, the most frequent ones are: ‘EEG’ (‘Electroencephalograms’) (occurring in 1,934 papers), ‘image’ (1,768), ‘detection’ (1,141), ‘segmentation’ (981), ‘fMRI’ (‘functional Magnetic Resonance Imaging’) (952), ‘interface’ (909), and ‘connectivity’ (898). We further adopted triangulation strategy to verify the choice and labels of the 30-topic model, using three other topic modeling techniques, that is, latent Dirichlet allocation (LDA) using variational expectation maximization (VEM) and Gibbs sampling, as well as latent semantic analysis (LSA). For all the four methods, the 30-topic model was found to be the best, ensuring the choice of the STM model with 30 topics. In addition, interpretations of the 30 topics for the four models were similar, ensuring the labels of the model. **Table 3** shows examples of topic modeling results for the four models. For example, regarding *Brain Image Processing*, several terms such as ‘MR’, ‘MRI’, ‘image’, and ‘segmentation’ appeared in the four models. As for *Brain-Computer-Interface*, all of the four models contained terms such as ‘interface’, ‘BCI’, ‘brain-computer’, and ‘computer’. For *Brain Disease*, relevant terms such as ‘AD’, ‘MCI’, ‘mild’, ‘impairment’, and ‘ASD’, were commonly found in the four models. For *Brain Tumor*, all of the four models contained terms such as ‘glioma’, ‘glioblastoma’, ‘grade’, ‘tumor’, and ‘brain’. For *Mental Disorder*, several terms such as ‘ASD’, ‘ADHD’, ‘disorder’, ‘autism’, ‘depression’, and ‘autism’, appeared in the four models.

**Table 3:** Interpretations of the topics fitted using STM, VEM, Gibbs sampling, and LSA.

*Brain Image Processing*	
STM	multi-atlas, segmentation, superpixel, c-means, deformable, MR-image, label, registration, inhomogeneity
VEM	image, brain, classification, feature, MRI, imaging, MR, transform, segmentation, detection
Gibbs sampling	image, segmentation, brain, MRI, MR, automatic, imaging, technique, c-means, MR-image
LSA	segmentation, image, MRI, MR, imaging, atlas, region, diffusion, clustering, registration
*Brain-Computer-Interface*	
STM	speller, MI—BCI, RSVP, ERRP, BCI, single-trial, brain-computer, imagery, p300, interface, MI
VEM	interface, BCI, brain-computer, signal, motor, system, performance, computer, movement, spatial
Gibbs sampling	interface, BCI, brain-computer, motor, signal, performance, computer, spatial, single-trial, p300
LSA	BCI, interface, computer, motor, imagery, brain, movement, spatial, p300, stimulus
*Brain Disease*	
STM	AD, MCI, amnestic, mild, MCI-C, alzheimer, dementia, PD, impairment, ADNI, atrophy
VEM	disorder, child, autism, spectrum, brain, ADHD, ASD, deficit, diagnosis, syndrome
Gibbs sampling	disease, alzheimer, cognitive, impairment, AD, mild, diagnosis, dementia, MCI, patient
LSA	AD, BCI, alzheimer, disease, MCI, impairment, mild, cognitive, diagnosis, dementia
*Brain Tumor*	
STM	metastasis, radiomic, glioma, glioblastoma, neuro-oncology, grade, GBM, survival, spectroscopic
VEM	tumor, glioma, patient, glioblastoma, survival, metastasis, grade, brain, cancer, high-grade
Gibbs sampling	tumor, glioma, patient, glioblastoma, brain, cancer, survival, grade, tumour, metastasis
LSA	tumor, glioma, feature, disorder, grade, glioblastoma, classification, spectroscopy, survival, meningioma
*Mental Disorder*	
STM	ADHD, MDD, first-episode, BD, SZ, ASD, schizophrenia, autism, psychotic, depression
VEM	disorder, child, autism, spectrum, brain, ADHD, ASD, deficit, diagnosis, syndrome
Gibbs sampling	disorder, patient, schizophrenia, depression, symptom, ADHD, deficit, bipolar, depressive, abnormality
LSA	disorder, autism, ADHD, ASD, attention, spectrum, child, deficit, hyperactivity, diagnosis

Abbreviations are displayed in **S3 Table**.

**Table 4** shows the 30-topic STM results, which includes the proportions in the whole dataset and developmental trends of the 30 topics, as well as the most discriminating terms, that is, frequent and exclusive terms with a high frequency-exclusivity (FREX) value [47] for each topic These prominent topics are divided into three proportion-based intervals (> = 4%, 3%-4%, and <3%), which are primarily the quartile and median values (rounded down to the nearest integers and merging the two lower quarters).

**Table 4:** The 30-STM results with the discriminating terms, topical proportions in the whole dataset, suggested topic labels, and topical developmental trends. The rows marked in dark grey are topics whose proportions are above 4%, those in light grey are topics whose proportions are between 3% and 4%, and those in white are topics whose proportions are below 3%.

Discriminating terms	%	Suggested topic	*trend*
vector, machine, SVM, support, kernel, feature, selection, classification, dimensionality, ELM, feature-selection, discriminative, classifier	7.28	*Classification Algorithms*	↑↑↑
EMD, IMF, multifractal, apnea, non-focal, ApEn, k-complex, sleep, entropy, wavelet, epileptic, REM, transform	6.35	*EEG Signals Analysis*	↑↑
multi-atlas, FCM, segmentation, superpixel, c-means, PVS, deformable, MR -image, contour, label, registration, inhomogeneity, IBSR	6.17	*Brain Image Processing*	↓
speller, CSP, SSVEP, MI—BCI, RSVP, ERRP, BCI, single-trial, brain-computer, imagery, p300, interface, MI	5.39	*Brain-Computer-Interface*	↓
AD, MCI, amnestic, AMCI, BVFTD, mild, MCI-C, alzheimer, dementia, PD, impairment, ADNI, atrophy	4.71	*Brain Disease*	↑
small-world, RSN, CNN, convolutional, network, graph-theoretical, granger, FC, node, deep, topological, topology, centrality	4.16	*Network*	↑↑↑
ADHD, MDD, first-episode, OCD, BD, REHO, SZ, ALFF, ASD, schizophrenia, autism, psychotic, depression	4.13	*Mental Disorder*	↑↑↑
bayesian, gaussian, mixture, markov, estimation, modeling, model, regression, inference, monte, sampling, GMM, carlo	4.01	*Statistical Modeling*	↓
CAD, GLCM, biogeography-based, computer-aided, CMB, texture, medical, co-occurrence, GEPSVM, curvelet, eigenbrain, landmark, image	3.96	*Computer-Aided Diagnosis*	↑↑
multivoxel, MVPA, scene, visual-cortex, ategory, categorization, representation, natural, decoding, pattern-analysis, identity, naturalistic, face	3.7	*Vision*	↓↓
brainmap, parcellation, insula, STS, subregion, insular, cingulate, empathy, social, amygdala, gyrus, connectivity-based, anterior	3.52	*Functional Connectivity*	↓
brainage, thickness, IQ, aging, morphometry, age, gray, gyrification, neuroanatomical, voxel-based, surface-based, GM, young	3.46	*Brain Development*	↑
music, band, emotion, theta, PLV, unpleasant, arousal, valence, affective, power, schizotypy, oscillation, synchronization	3.46	*Emotion*	↑↑
synapsis, memristor, neuromorphic, memristive, reservoir, STDP, SNN, self-organization, latching, synaptic, spiking, associative, neuron, HTM	3.44	*Nervous System*	↓
dictionary, swarm, particle, sparse, ICA, removal, sparsity, inverse, denoising, optimization, PSO, separation, beamformer	3.41	*Optimization Algorithms*	↑
reward, FRN, aversive, reinforcement, dopamine, striatum, ganglion, valuation, tegmental, decision-making, BG, reversal, punishment	3.15	*Decision-Making*	↓↓
exoskeleton, upper-limb, extremity, brain-machine, BMI, brain-robot, flexion, movement, finger, rehabilitation, hand, arm, TDCS	2.89	*Motor & Robot*	↓
driver, drowsiness, wearable, drowsy, consumer, SOC, driving, fatigue, aesthetic, workload, neuro-fuzzy, vigilance, ANFIS	2.83	*Fatigue Driving*	↑↑↑
metastasis, radiomic, PTSD, RCBV, glioma, glioblastoma, neuro-oncology, non-enhancing, multiforme, grade, GBM, survival, spectroscopic	2.78	*Brain Tumor*	↑
TBI, preterm, cost-effectiveness, TCD, infant, hypoxic-ischaemic, aneurysm, neonatal, traumatic, injury, gestation, HIE, prehospital	2.7	*Infant*, *Fatal & Child*	↓
tensor, DTI, tractography, anisotropy, diffusivity, microstructural, peduncle, capsule, HARDI, DMRI, diffusion, cartilage, microstructure	2.54	*Brain Structure*	↓
neglect, visual-search, attentional, attention, microstate, orienting, saliency, selective, visuospatial, search, RTMS, gaze, top-down	2.46	*Attention & Vision*	↓↓↓
PET/MRI, MR-AC, GTV, penumbra, attenuation, infarct, vessel, PET/MR, F-18-FET, positron, SUV, PET/CT, emission	2.41	*Brain Imaging*	↓
lexical, verb, p600, MMN, semantic, word, sentence, syntax, syntactic, RHD, ERP, reading, classifier-noun	2.21	*Semantic Cognition*	↓↓↓
TLE, STN, IED, IEEG, neurostimulation, focal, epilepsy, mesial, DBS, epileptiform, SEEG, epileptogenic, pre-surgical	2.09	*Epilepsy*	↑
methylation, microarray, genome-wide, epigenetic, mirna, BDNF, GWAS, single-nucleotide, microrna, galectin, mitotic, histone, methyltransferase	1.68	*Gene*	↓↓
HIV, meningitis, virus, TDP-43, neurofibrillary, hypomyelination, CJD, TLR, parasite, aseptic, retinopathy, antiretroviral, NFT	1.42	*Virus & Pathology*	↓↓↓
speech, tinnitus, vowel, cochlear, pitch, prosody, sensorineural, dysarthria, stuttering, monolingual, sound, hearing, auditory	1.28	*Phonological Cognition*	↓
near-infrared, FNIRS, anesthesia, infrared, vegetative, propofol, sevoflurane, BI, HBO, DOA, consciousness, optical, depth	1.27	*Near-Infrared Spectroscopy*	↑
metabolomic, blood-brain, BBB, NMF, PNES, influx, microscopy, spectrometry, DCE-MRI, mass, factorization, permeability, barrier	1.12	*Molecule*	↓↓

Topics are ranked by proportion in a descending order. %: topic proportions in the dataset (with the *θ* matrix estimated by STM, where *θ*_*ij*_ (*i* = 1,2,…6317, *j* = 1,2,…30) denotes the proportion of document *i* allocated to topic *j*. Proportion of each topic obtained by summing up *θ*_*ij*_ by topic). Abbreviations are shown in **S3 Table**. ↑(↓): increasing (decreasing) trend but not statistically significant (*p* > 0.05); ↑↑(↓↓), ↑↑↑(↓↓↓), ↑↑↑↑(↓↓↓↓): statistically significant increasing (decreasing) trend (*p* < 0.05, *p* < 0.01, and *p* < 0.001, respectively)

Of the 30 prominent topics, 25 are specific subjects concerning human brain research, which are *Brain Development*, *Phonological Cognition*, *Nervous System*, *Brain Structure*, *Semantic Cognition*, *Brain Image Processing*, *Decision-Making*, *Epilepsy*, *EEG Signals Analysis*, *Molecule*, *Brain-Computer-Interface* (*BCI*), *Motor & Robot*, *Brain Disease*, *Functional Connectivity* (*FC*), *Brain Tumor*, *Brain Imaging*, *Vision*, *Emotion*, *Infant*, *Fatal & Child*, *Virus & Pathology*, *Attention & Vision*, *Gene*, *Mental Disorder*, *Fatigue Driving*, and *Near-Infrared Spectroscopy*. These topics account for 81.13% of the whole dataset. Another four topics, namely, *Computer-Aided Diagnosis*, *Classification Algorithms*, *Statistical Modeling*, and *Optimization Algorithms*, are about general technologies, algorithms, or methods. They account for 18.87% of the whole dataset. The remaining topic, *Network*, is method-related or brain-structure-related. It accounts for 4.16% of the whole dataset.

The top five topics having the highest proportions in the dataset are: *Classification Algorithms*, *EEG Signals Analysis*, *Brain Image Processing*, *Brain-Computer-Interfaces*, and *Brain Disease*. Their developmental trends, correlations, and distributions among countries/regions and research institutes are investigated in the following sections.

### Topical trends

**Table 4** also shows the results of the MK test for the 30 topics. Seven topics, namely *Classification Algorithms*, *Computer-Aided Diagnosis*, *EEG Signals Analysis*, *Network*, *Emotion*, *Mental Disorder*, and *Fatigue Driving*, exhibit statistically significant increasing trends. Another seven topics, namely, *Semantic Cognition*, *Decision-Making*, *Molecule*, *Vision*, *Virus & Pathology*, *Attention & Vision*, and *Gene*, show statistically significant decreasing trends. The remaining 16 topics have no statistically significant trends.

**Fig 2:**
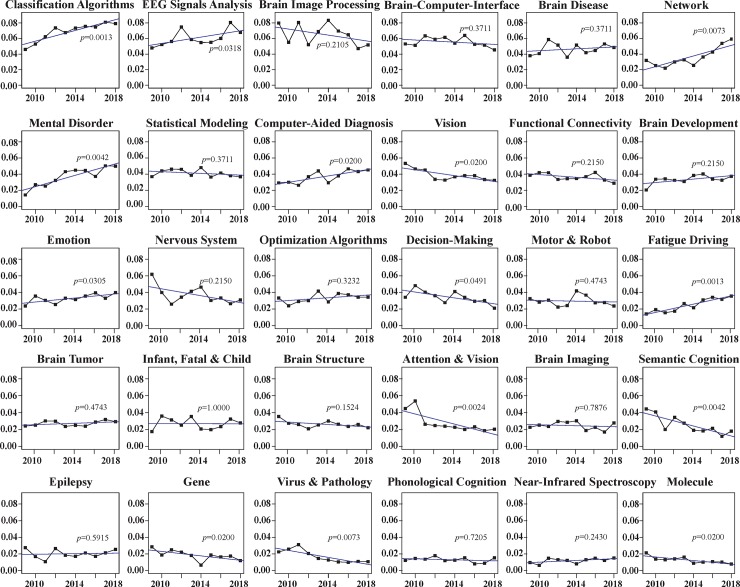
Annual trends of the identified topics (2009–2018).

**Fig 2** visualizes these trends by showing the varying prevalence of each of the 30 topics over the past decade in the whole dataset. In each of the 30 plots, the black line is the actual distribution of a topic, with black dots indicating annual topic proportions in the dataset, and the blue line is the cubic (or Hermite) spline interpolation of the annual topic proportions, in which the spline used is from Forsythe, Malcolm, and Moler [48]. The *p* value in each panel comes from the Mann-Kendall trend test.

### Topical correlations

**Fig 3:**
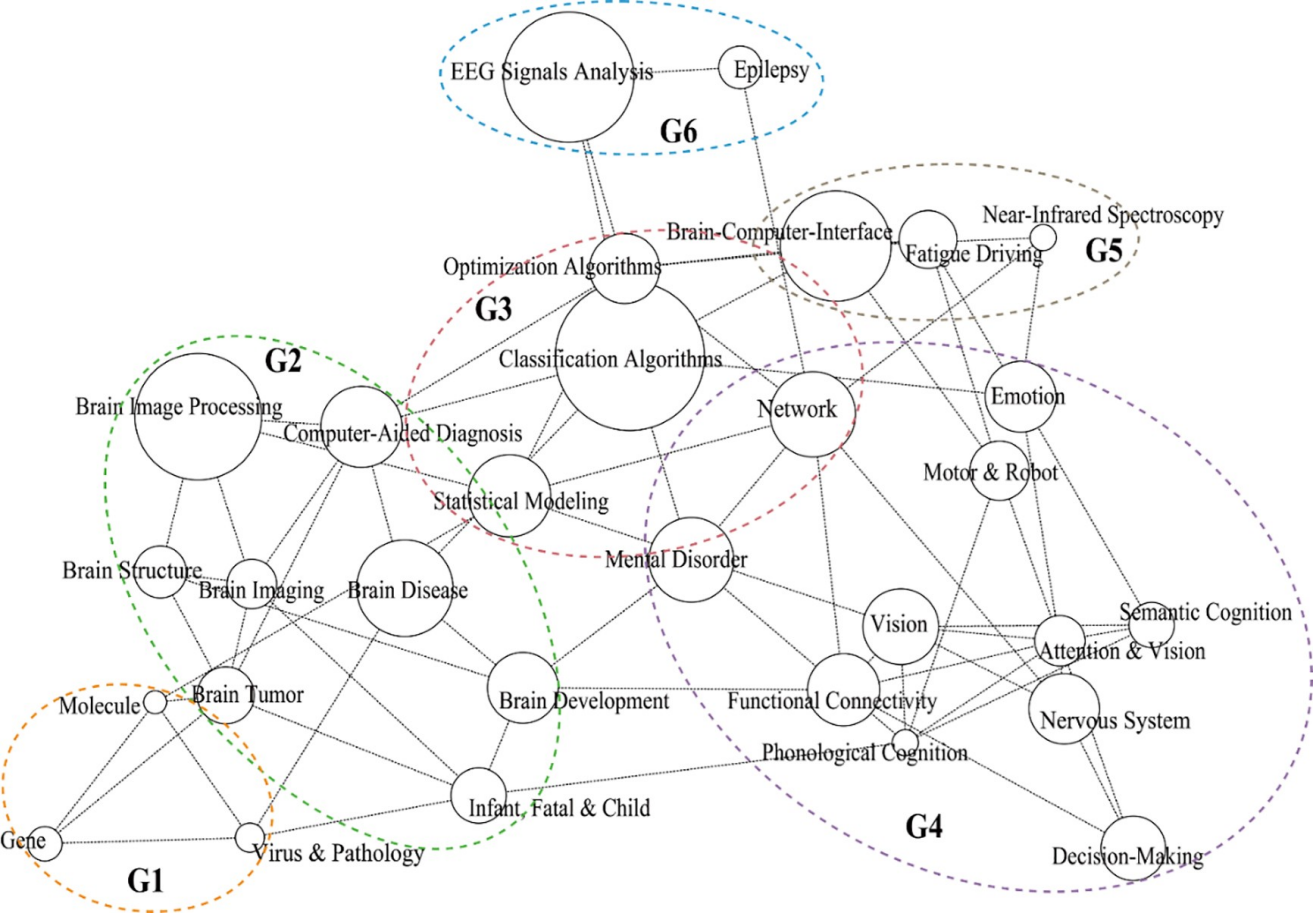
Graphing positive correlations between the 30 identified topics.

**Fig 3** shows the topic correlations based on a semi-parametric Gaussian procedure implemented using the R package *huge* [49]. In the figure, each topic is represented by a circle, the size of which is proportional to the topic proportion in the whole dataset. Topics connected by a dotted line indicate that they are more likely discussed within a paper, that is, the two topics are positively (>0) correlated. Correlation is calculated using a non-paranormal conversion of the topic proportions with the adoption of semiparametric Gaussian copulas. A shorter link between two topics means a higher correlation between the two. Topics that are negatively (≤0) correlated are not connected. Colored ellipses are added to point readers toward the six emergent and distinct clusters (marked by G1 to G6).

Within the cluster G1 are three topics: *Gene*, *Virus & Pathology*, and *Molecule*. G2 includes eight topics, mostly brain-related, such as *Brain Tumor*, *Brain Structure*, *Brain Imaging*, *Brain Image Processing*, *Brain Development*, and *Brain Disease*. G3 focuses on methods or algorithms, including *Classification Algorithms*, *Statistical Modeling*, *Optimization Algorithms*, and *Network*. G4 pertains to cognition-related topics, such as *Phonological Cognition*, *Semantic Cognition*, *Attention & Vision*, *Vision*, and *Emotion*. G5 includes *Brain-Computer-Interface*, *Fatigue Driving*, and *Near-Infrared Spectroscopy*. The cluster G6 at the top of the figure comprises two topics: *EEG Signals Analysis* and *Epilepsy*. These broad clusters provide a comprehensive profile of the emphases in AI-assisted human brain research from the year 2009 to 2018.

### Topic distributions across top countries/regions and institutes, as well as topic distribution by year

**Fig 4:**
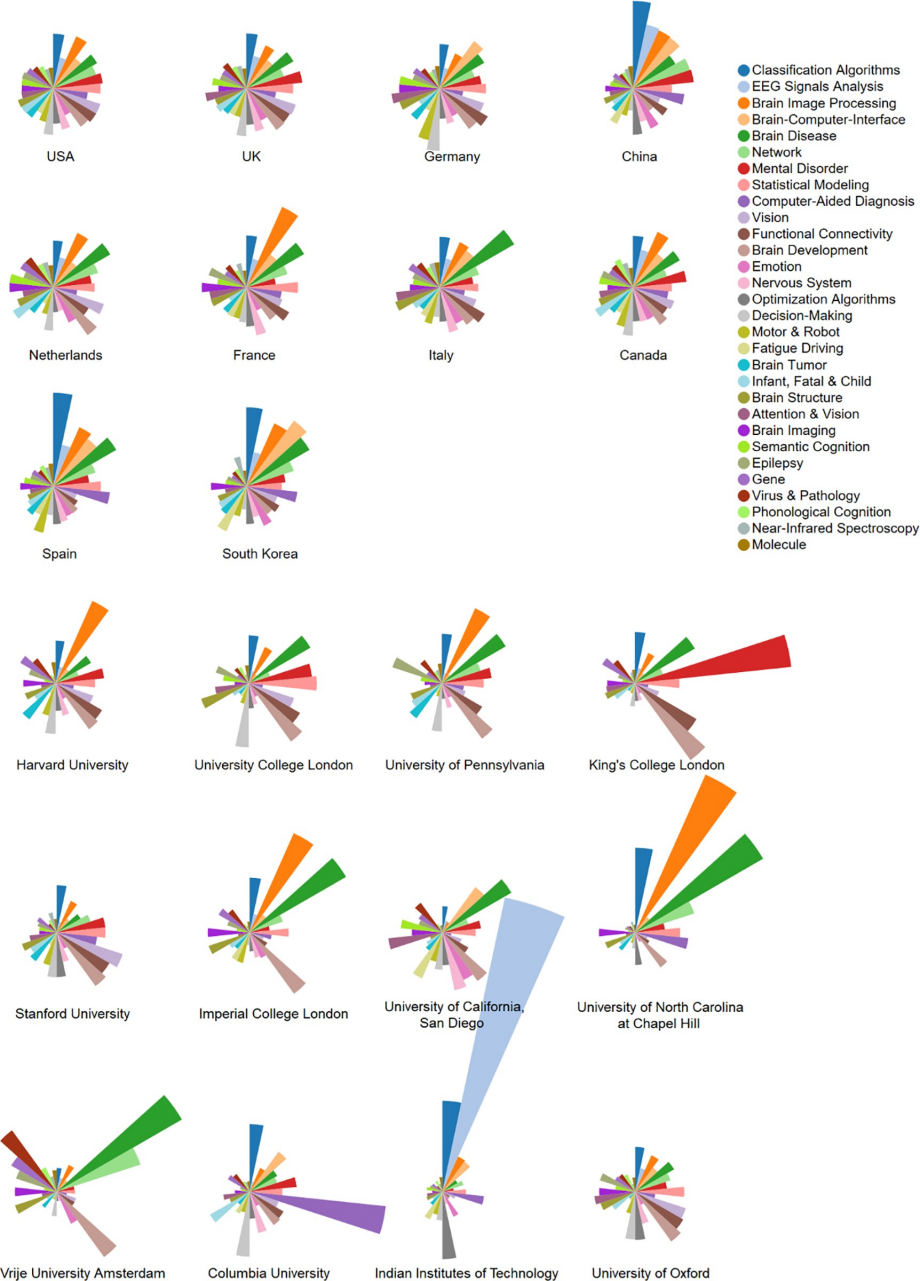
Topic proportion distributions of the influential countries/regions and institutes in AI-assisted human brain research ranked by h-index.

Influential countries/regions and research institutes in AI-assisted human brain research were identified in terms of the quantity of relevant papers, citations of those papers, and topical advantages. **Fig 4** illustrates the topical distributions among the research units ranked by *h*-index, based on their topical proportion metrics. Based on the topical proportion metric of prolific countries/regions (or institutes) in json format, we used the graphing tool Cluster Purity Visualizer to obtain a basic distribution graph. All distributions show great diversities. **S1 Fig** and **S4 Table** provide the detailed paper and citation counts of those countries/regions and institutes.

As for countries/regions (see the upper panel of **Fig 4**), China, Spain, and South Korea are more productive in *Classification Algorithms*, and France is especially productive in *Brain Image Processing*. In addition, the research enthusiasm for *Brain Disease* on the part of Italy and *Brain-Computer-Interface* on the part of South Korea are worth noting, since the proportions (9.96% and 9.27%, respectively) of these topics in those countries are the highest among all the listed countries/regions.

As for institutes (see the lower panel of **Fig 4**), King’s College London, Columbia University, Indian Institutes of Technology, and University of North Carolina at Chapel Hill are more productive in *Mental Disorder*, *Computer-Aided Diagnosis*, *EEG Signals Analysis*, and *Brain Image Processing*, respectively. Vrije University Amsterdam and University of North Carolina at Chapel Hill are more productive in *Brain Disease*.

**Fig 5:**
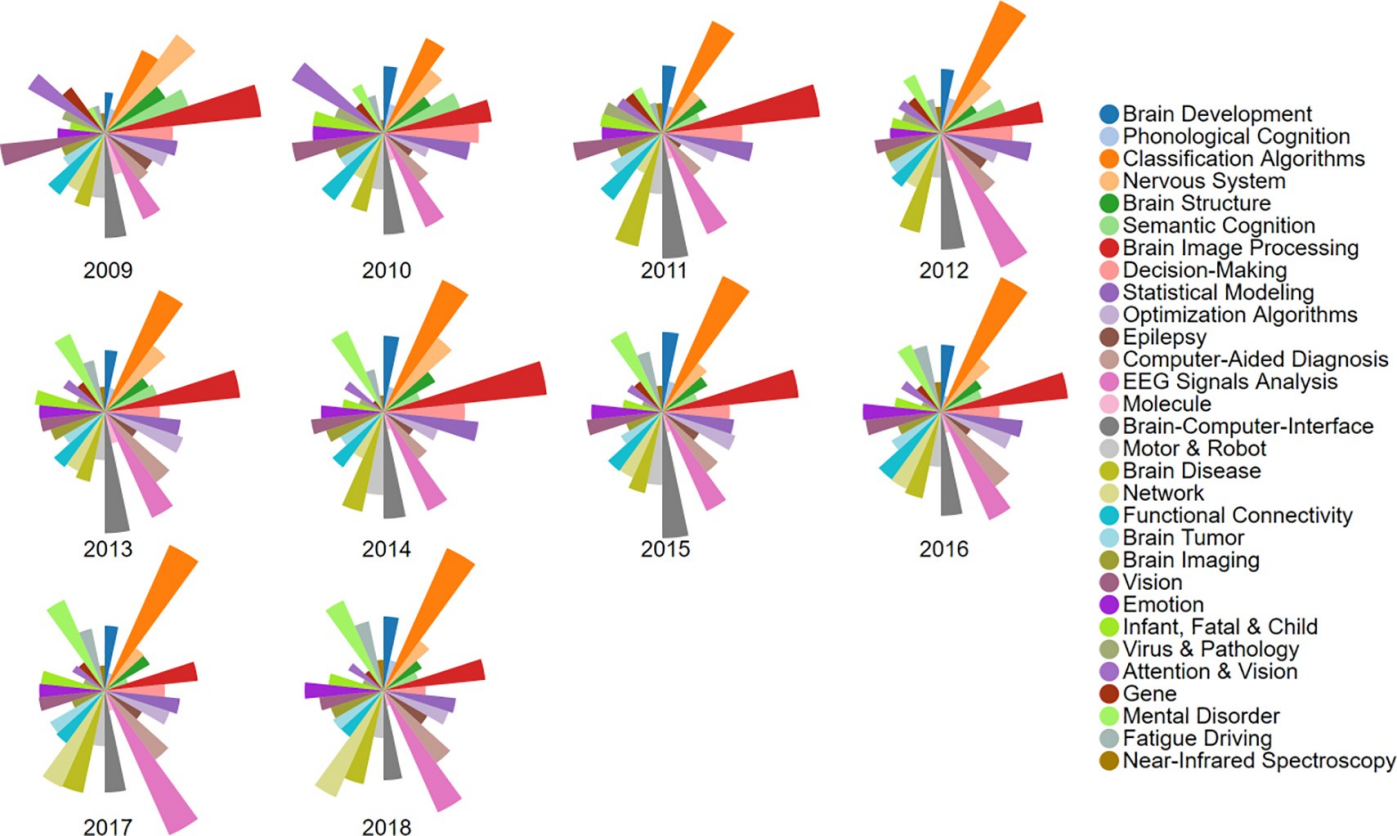
Topic proportion distributions by year.

**Fig 5** visualizes the topic proportion distributions annually in AI-enhanced human brain publications. Generally, during the period 2009–2018, the community has paid balanced attention to most of the research topics. The results clearly depict the dominated topics for each year. For instance, *Brain Image Processing* and *Classification Algorithms* were the most focused topics for most of the years, particularly in previous few years for *Brain Image Processing* and in recent few years for *Classification Algorithms*. Some topics have decreased in research. For example, *Attention & Vision* received more attention in 2009 and 2010 as compared to following years. *Vision* was studied more during the period 2009–2012 comparing to the following years. *Decision-Making* was studied most mainly during the period 2009–2014. Some topics have increased in research. For example, *Mental Disorder* had received more attention from authors in recent years, particularly during the period 2013–2018, as compared to former few years. *Network* was focused more during the period 2015–2018, comparing to the former few years.

### Topic differences in funding and international collaboration

**Fig 6:**
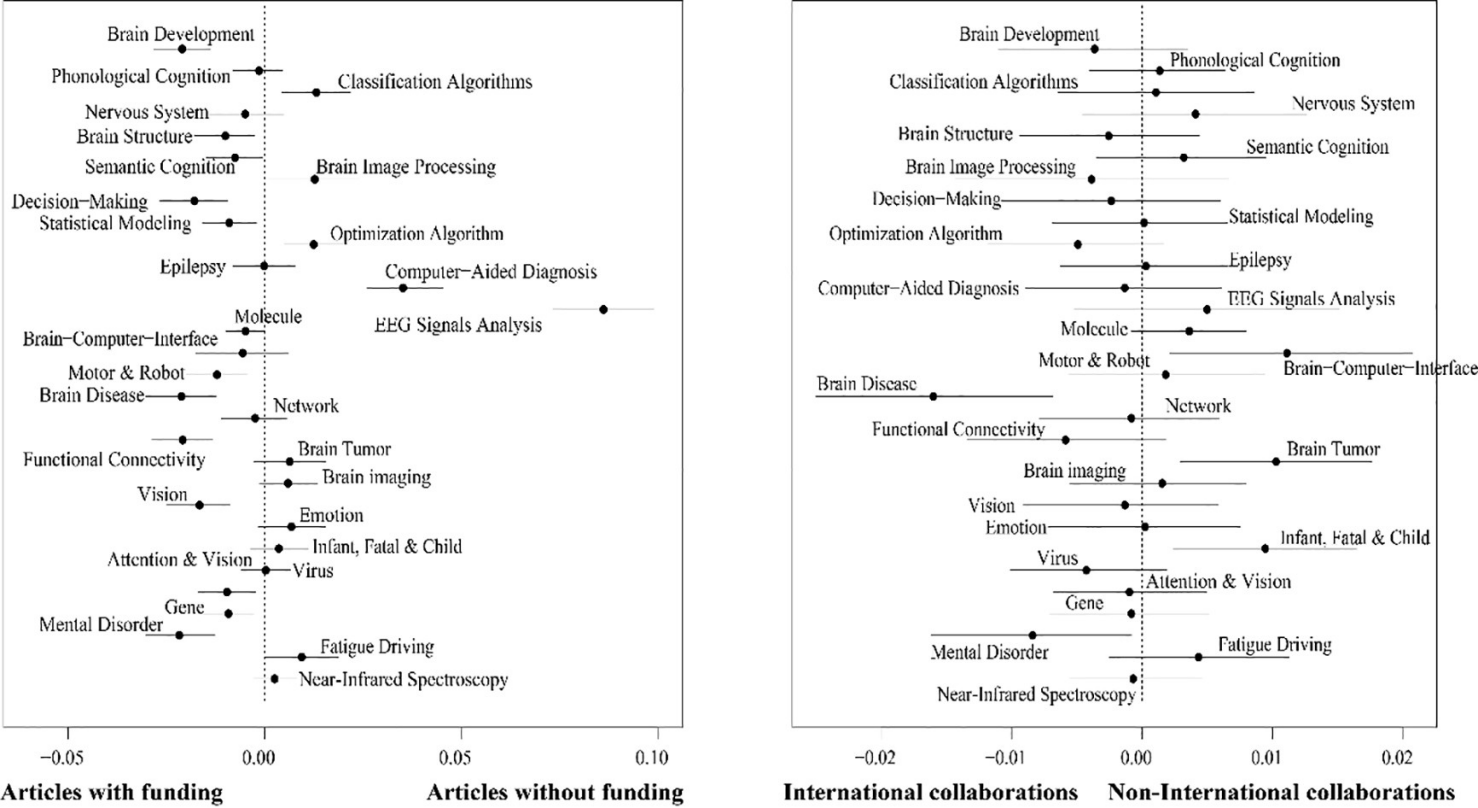
Effects of funding and international collaboration on topic proportions. Differences between papers with and without funding (a), as well as papers with and without international collaboration (b).

We compared academic concerns on the AI assisted human brain research based on the subsets related to funding and international collaboration, as shown in **Fig 6**. Values in the figure were calculated using linear regression, where the proportion of each topic in a paper was used as the dependent variable while the explanatory variable was binary, specifying whether or not the paper was with funding and international collaboration. Effects of funding on topic proportions are shown in **Fig 6(A)**, where topics on the left are discussed more in funded papers. Twelve topics, namely *Brain Development*, *Brain Structure*, *Semantic Cognition*, *Decision-Making*, *Statistical Modeling*, *Motor & Robot*, *Brain Disease*, *Functional Connectivity*, *Vision*, *Attention & Vision*, *Gene*, and *Mental Disorder* appeared significantly (*p* < 0.05) more in funded papers, while five topics, namely *Classification Algorithms*, *Brain Image Processing*, *Optimization Algorithms*, *Computer-Aided Diagnosis*, and *EEG Signals Analysis* appear significantly more in non-funded research. As for 13 other topics showing no significant differences between funded and non-funded. Likewise, differences of topic prevalence between papers with and without international collaboration are shown in **Fig 6(B)**. International collaboration has more neutral effects. Only two topics, *Brain Disease* and *Mental Disorder*, are more often seen in papers with international collaboration, while *Brain-Computer-Interface*, *Brain Tumor*, and *Infant*, *Fatal & Child* are more frequently discussed in papers without international collaboration.

## Discussions

### Most representative study for each topic

We here provide the most preventative paper for each topic. For *Brain Development*, Hoekzema et al. [50] aimed to investigate if there were signs of a sex-atypical brain development in gender dysphoria. They first quantified regional neural gray matter volumes in 55 female-to-male and 38 male-to-female adolescents, 44 boys and 52 girls without gender dysphoria. They then applied univariate and multivariate approaches for data analyses. For *Phonological Cognition*, by assessing spoken language comprehension in non-speaking children with severe cerebral palsy, Geytenbeek et al. [51] explored the relationship between motor type and disability using multiple linear regression method. For *Classification Algorithms*, Siuly and Li [52] presented an innovative approach to classify multiclass EEG signals, which involved the adoption of optimum allocation algorithm for selecting representative samples. For *Nervous System*, Yang et al. [53] conducted experiments and simulations by adopting second-order memristors to highlight the suppression triplet-spike-timing dependent plasticity learning rule. For *Brain Structure*, Lancione et al. [54] assessed how tissue structural orientation affected quantitative susceptibility mapping reliability and provided principles for identifying voxels where magnetic susceptibility (chi) measures were mainly affected by spatial orientation effects. For *Semantic Cognition*, Zhou et al. [55] investigated the temporal neural dynamics of semantic integration processes at various levels of syntactic hierarchy while reading Chinese sentences. For *Brain Image Processing*, Yang et al. [56] presented an innovative brain tissue segmentation approach in magnetic resonance images using neighborhood spatial information as a basis with the combination of classical fuzzy C-means clustering and Markov random field approaches. For *Decision-Making*, Park et al. [57] examined if the releases of norepinephrine and dopamine in the ventral and dorsolateral bed nucleus of the stria terminalis correlated with reward learning during intracranial self-stimulation. For *Statistical Modeling*, Soch and Allefeld [58] presented an innovative statistical parametric mapping toolbox for assessing, comparing and selecting general linear models for analyzing fMRI data. For *Optimization Algorithms*, Parsopoulos et al. [59] investigated the potential of particle swarm optimization (PSO) and unified PSO for addressing magnetoencephalography (MEG) issues. For *Epilepsy*, Jeong et al. [60] aimed at devising a novel clustering approach for MEG interictal spike sources and identifying its potential value in adult epilepsy patients with cortical dysplasia. For *Computer-Aided Diagnosis*, Kathirvel and Batri [61] proposed an innovative fully-automated computer-assisted approach to detect brain tumor with the use of co-active adaptive neuro-fuzzy inference system classifier. For *EEG Signals Analysis*, Li et al. [62] proposed an innovative hybrid automated sleep stage scoring method called HyCLASSS with the basis of single channel EEG. For *Molecule*, to enable comparing blood-brain barrierinflux (BBB) results of peptides directly, Stalmans et al. [63] proposed an innovative classification approach and unified response for BBB transport of peptides. For *Brain-Computer-Interface*, Schettini et al. [64] proposed and evaluated a novel approach for the automated recalibration of the classifier’s parameters. For *Motor & Robot*, Kraus et al. [65] examined changes of corticospinal excitability with transcranial magnetic stimulation in 13 right-handed healthy participants. For *Brain Disease*, Yu et al. [66] aimed at identifying the ideal combination of MRI, [F-18]-fluorodeoxyglucose positron emission tomography, and cerebrospinal fluid biomarkers for predicting transformation from amnestic mild cognitive impairment to Alzheimer's disease dementia. For *Network*, Wang et al. [67] investigated the differences in the dynamic brain network during resting and visual stimulation statuses in a task-positive sub-network, task-negative sub-network, and whole-brain network. For *Functional Connectivity*, Deen et al. [68] adopted resting-state FC MRI for parcellating the human insular lobe with the basis of FC patterns clustering. For *Brain Tumor*, Blüml et al. [69] examined whether differences existed in metabolite concentrations measured by magnetic resonance spectroscopy between molecular sub-groups of medulloblastoma. For *Brain Imaging*, Mourik et al. [70] aimed at validating in vivo the accuracy of a reconstruction-driven partial volume correction by considering the point spread function of imaging systems. For *Vision*, aiming at studying shapes extraction using temporal incorporation of successive partial shape views, Orlov and Zohary [71] showed participants the artificial shapes moving behind a narrow vertical or horizontal slit. For *Emotion*, Petrantonakis and Hadjileontiadis [72] aimed at providing an innovative approach to evaluate the emotion elicitation processes within an EEG-driven emotion recognition system. For *Infant*, *Fatal & Child*, Goto et al. [73] proposed an easy-to-use and generally applicable bedside instrument to predict outcomes in children after cardiac arrest. For *Virus & Pathology*, Hiar et al. [74] assessed epidemiological, clinical, and laboratory features of enterovirus infections of central nervous system in children younger than 15 years. For *Attention & Vision*, with the use of human MEG, Bartsch et al. [75] examined whether effects of global feature-based attention were preserved by manipulating the strength and consistency of spatial focusing to the target. For *Gene*, with the use of unsupervised hierarchical clustering, Perez-Magan [76] identified gene expression profiles and candidate markers related to original and recurrent meningiomas. For *Mental Disorder*, Guo et al. [77] adopted fractional amplitude of low-frequency fluctuation to examine regional alterations of the default mode network in unaffected siblings of schizophrenia patients during resting. For Fatigue Driving, Li and Chung [78] proposed an innovative context-aware brain machine interface system for detecting driver drowsiness at early stage. For *Near-Infrared Spectroscopy*, Hernandez-Meza et al. [79] examined the potential of functional near infrared spectroscopy to monitor anesthetic effects on prefrontal cortex.

### Topical proportions and trends

The topical intervals in **Table 4** and the developmental trends in **Fig 2** clarify different groups of topics with different degrees of prominence. First, there are eight frequently discussed topics in the dataset, each with a proportion over 4% and accounting for 42.21% in total. Four of them, namely, *Classification Algorithms*, *EEG Signals Analysis*, *Network*, and *Mental Disorder*, show significantly increasing trends. This indicates that these four topics have not only received much attention (21.93%) over the past decade, but they will also probably continue to be the research foci in the near future. By contrast, the other four topics, i.e., *Brain Image Processing*, *Brain-Computer-Interface*, *Brain Disease*, and *Statistical Modeling*, have no significant tendencies. This suggests that although those topics received great interest over the past decade (in total accounting for 20.29% of the whole dataset), especially in the previous few years, it is difficult to tell whether their developing momentums would maintain in the near future.

Second, there are eight topics with proportions between 3% and 4% and together accounting for 28.10% of the whole dataset. Only two of them, *Computer-Aided Diagnosis* and *Emotion*, show significantly increasing trends. These two topics have consistently been the research foci over the past decade, and it is entirely possible for them to continue to be ‘hot’ issues in the near future. By contrast, research interests in the other six topics, especially *Decision-Making* and *Vision*, have declined over the past decade; it is likely that fewer and fewer studies in those topics will be conducted in the near future.

Third, the remaining 14 topics have low proportions (each below 3% and accounting for 29.68% in total). Among them, only *Fatigue Driving* shows a significantly increasing trend. This topic is at its developmental stage, demonstrates great research potential, and will probably gain more interest and attention in the near future. Five topics, namely, *Attention & Vision*, *Semantic Cognition*, *Gene*, *Virus & Pathology*, and *Molecule*, show significantly decreasing trends. This suggests that not only have they attracted little attention in the past decade, but they are also likely to be less popular in AI-assisted human brain research in the near future.

Examining the detailed developmental trends of different topics in **Fig 2** also reveals different degrees of interest and attention obtained by those topics. Frist, several topics received increasing attention throughout the whole studied period, e.g., *Classification Algorithms*, *Mental Disorder*, *Fatigue Driving*, and *Computer-Aided Diagnosis*. The steady growth of *Classification Algorithms* indicates the dominant popularity of applying such algorithms, a major AI technique, to human brain research throughout recent years. Classification of neuroimaging data for diagnosis of brain diseases or mental illnesses is a main goal of neuroscience research and clinical treatment. Accumulating evidence indicates that applying classification algorithms to neuroimaging measures is valuable for developing diagnostic and prognostic prediction tools in psychiatry. Regarded to be one of the main causes of traffic accidents worldwide, *Fatigue Driving* has been an attractive subject in the recent decades, and to effectively detect driver fatigue is of significance to public health and safety. It is expected that these topics are and will remain prominent in future research.

Second, certain topics start to show increasing trends after a specific year within the past decade. For example, *Network* began to gain increasing attention around 2014, and its increasing speed (reflected by the slope of the curve) is the highest among the topics showing significantly increasing trends. Network-based techniques, such as artificial neural networks, excel at analyzing challenging datasets and serve as exceptional tools to support decision-making in clinical treatment. Complex networks also serve as a repetitive problem in neuroimaging data analysis [80].

Third, several topics exhibit decreasing trends at certain time in the past decade. For example, *Attention & Vision* started to show a decreasing trend after 2010, and *Virus & Pathology* began to receive less attention after 2011. Topics such as *Brain Image Processing*, *Brain-Computer-Interface*, *Nervous System*, *Motor & Robot*, and *Decision-Making* started to show decreasing tendencies after 2014. The decreasing trend became more explicit for *Functional Connectivity* after 2016. Two other topics, namely, *Vision* and *Semantic Cognition*, demonstrate continuously decreasing trends throughout the whole decade.

Finally, as for the other topics not showing statistically significant trends, some topics, such as *Brain Image Processing* or *Brain-Computer-Interface*, remained popular throughout the decade, whereas other topics, such as *Phonological Cognition*, *Near-Infrared Spectroscopy*, or *Brain Tumor*, were less so throughout the decade.

### Topical correlations

Topical correlations in **Fig 3** demonstrate the close and mutual influence between AI and human brain research. On the one hand, applications of AI technologies in human brain research are ubiquitous, necessary, and important. AI technologies comprise the core of computational neuroscience, and they are able to inspire and stimulate brain research. As in **Fig 3**, the method-related cluster lies in the central position, having the most links with the other topics. In particular, *Classification Algorithms* is a popular technology widely used in many research topics, including *Brain-Computer-Interface*, *Mental Disorder*, *Brain Disease*, *Computer-Aided Diagnosis*, and *EEG Signals Analysis*. Classification of mental tasks and related EEG signals is one of the key issues and challenges of EEG-based BCI [81]. Classification technologies have been applied in diagnosis and detection of mental disorders such as depression [82]. Classification analysis of brain imaging helps recognize abnormal activities in brain functionality [83]. EEG signals classification is also essential for diagnosing and treating brain diseases [84]. In addition, classification of emotion are widely concerned by scholars, not only within biomedical field, but also in social science research (e.g., [85–88]).

Besides, the close topical correlations between *Network* and *Mental Disorder*, *Near-Infrared Spectroscopy*, *Epilepsy*, and *Functional Connectivity* indicate where the network technologies are being applied and improved. For example, as simplified representations of structural and functional interactions, brain connectivity networks have been adopted for diagnosing and classifying neurodegenerative diseases [89]. Many studies attempt to develop detailed toolboxes to enhance innovative and comprehensive brain connectivity analysis. Many online, interactive platforms have become available for brain network analysis, e.g., the UCLA Multimodal Connectivity Database [90]. A few studies also demonstrate the diagnostic utility of network-related analyses in mental disorders.

In addition to these network-based applications, network theory serves as an intuitively attractive framework to investigate relations among interconnected brain regions (structural connectivity) and mechanisms (functional connectivity), as well as their relevance to behaviors. The network models used in neuroscience have extended this field “from data representations to first-principles theory, from biophysical realism to functional phenomenology, and from basic descriptions to coarse-grained approximations” (p.1) [91]. These extensions have brought forth better understanding about the structure, function, and development of human brain.

On the other hand, neuroscience offers rich sources of inspirations for novel AI technologies which are independent of and complementary to the mathematical and logic-driven approaches and idea dominance in traditional AI approaches. For example, artificial neural networks were originally inspired from the architecture of neurons in the brain, and neuroscience provided the initial guidance with respect to the architectural and algorithmic restrictions, which contributed to the success of the applications of neural networks in AI. Ever since the origin of artificial neural networks, many related technologies have been inspired, developed and fueled by the continuing development of brain research. AI has been revolutionized by significant progresses in neural-networks-related approaches over the past few years. For example, the convolutional neural networks integrate a number of canonical hallmarks of neural computations [92], which were a direct inspiration of single-cell recordings from mammalian visual cortex [93].

In addition, a variety of neural network technologies have been modified, in combination with other technologies, such as classification, to fulfill specific research needs. For example, a multi-layer perceptron classification approach based on neural networks was presented to support diagnosis of epilepsy [94]. An ‘anesthesia’-‘awareness’ discriminating system was proposed based on a neural network classifier and Granger causality features [95].

### Topical distributions in research units and collaborations of countries/regions

**Fig 4** reveals which countries/regions and institutes have the most influence on AI-assisted human brain research as a whole, or in specific topic(s) in the past decade. For example, the topical distributions of the USA, UK, and Canada are similar; compared to the other countries/regions, they are more balanced regarding almost every aspect of AI-assisted human brain research. The topical distributions of China, Spain, and South Korea are similar, all having a greater focus on the topics having relatively higher proportions. In particular, China can be regarded as an influential country in AI-assisted human brain research, due to its comparatively wider coverage of *Classification Algorithms*, *EEG Signals Analysis*, *Brain Image Processing*, *Brain-Computer-Interface*, *Network*, and *Mental Disorder*. China also has the highest proportions for almost all the seven topics demonstrating significantly increasing trends, followed by Spain and South Korea. This reveals the fact that although having fewer research outputs than the USA, these three countries are promoting the development of those seven topics. In addition, this quantitative analysis also illustrates the research strength of each country, in one or more topics. For example, South Korea is highly influential in research on *Brain-Computer-Interface*.

Similar insights can be drawn from the topical distributions in research institutes. It is worth highlighting that the topical strengths of some institutes are extremely significant. For example, Indian Institutes of Technology is more influential for *EEG Signals Analysis* research, and King’s College London for *Mental Disorder* research.

Diversity of disciplines and topics in countries/regions and institutes indicates that more effective AI-assisted human brain research relies on inter-regional, inter-institutional, and interdisciplinary collaborations. Such collaboration can incorporate the strengths of different research units or disciplines to overcome challenges and advance the whole field.

The network-based investigation on the collaborations in AI-assisted human brain research has shown that countries or institutes with similar research foci tend to collaborate more (see **S2 Fig**). To better understand the importance of different countries/regions in these collaborations, we adopted the approach of network analysis and calculated four typical centrality measures (i.e., degree, closeness, betweenness, and eigencentrality) of the top 20 most influential countries/regions involved in the network (see **S5 Table**). Degree-based centrality [96] reflects nodes’ relative dominance in a network. Closeness measures nodes’ centrality in terms of information transmission [97]. Node betweenness is another index measuring the importance of a node in controlling information transmission in a network [98]. Eigencentrality reflects the influence a node has on the whole network; if a node is pointed to by many nodes that also have high eigencentrality scores, the node also has a high eigencentrality score [99].

As in **S5 Table**, the USA dominates in all the four measures, indicating its overall importance and centrality in the collaboration network. UK is ranked the second by three measures except eigencentrality, by which Italy is ranked the second. Close collaborators of Italy include: the USA (collaborating in 53 papers), UK (51), Germany (36), and France (23), all having good performances in the four centrality measures. Performance of China is also worth noting in terms of degree (ranked the third), closeness (the first), betweenness (the third), and eigencentrality (the fourth). Close collaborators of China include: the USA (collaborating in 297 papers), UK (56), Australia (45), South Korea (36), Canada (31), and Japan (28).

These network-based findings and topical distribution results can promote and guide future collaborative investigations in AI-assisted human brain research.

### Topics that lack sufficient attention

Despite these findings, there are essential topics that deserve more attention from AI-assisted human brain research. For example, it is acknowledged that AI has brought forth many theoretical contributions to the interdisciplinary field of cognitive science [100], however, as a fundamental brain function widely studied in cognitive science, neuroscience, and psychology [101], the coverage of consciousness related terms in our dataset is small; e.g., ‘awareness’ and ‘conscious’ only appear in 55 and 45 papers, respectively. Language, as a complex, high-level brain function, is another ‘hot’ topic in human brain research, psychology, and linguistics, yet the coverage of related terms remains scarce; ‘language’ only appears in 24 papers. AI has already achieved great advancements in language related fields, such as natural language processing, yet AI-assisted human brain research seems not paying enough attention to these brain functions.

Intrinsic connectivity networks, especially the default-mode networks [102], and relations between these networks have been intensively investigated in cognitive neuroscience. Based on these brain structures and EEG signals, AI technologies, such as network analysis and classification algorithms, can help identify a conscious or unconscious brain and diagnose related diseases. Given detailed datasets concerned with human brain connectivity, AI can also generate useful clues on how fundamental (e.g., consciousness) and advanced (e.g., language) brain functions are possible via activation and connection of different parts of the brain, thus contributing to the general discussion of how intelligence emerges.

In addition, although classification algorithms are currently the main AI technologies applied in AI-assisted human brain research, other useful AI technologies remain limited in human brain research applications. For example, AI has proven values in health prediction, yet there are few studies that attempt to use structural or functional connectivity of human brain to temporally predict the degrees of high-level brain functions, such as reading [103]. Although AI-based longitudinal prediction has promoted related fields such as psychology or psychiatry [104], there is a dearth of research applying AI technologies to longitudinal brain imaging data to predict changes in psychological or neurological status of a person, e.g., degeneration of brain functions or progression of brain-related diseases.

### Latest trends in AI-enhanced human brain research

Latest trends in AI-enhanced human brain research are presented here to bring insights into what is happening in the research field. Latest trends in the applications of deep learning techniques in the AI-enhanced human brain research should be highlighted, which is covered within the topic *Network*. For example, O’Shea et al. [105] proposed an innovative deep-learning classifier for seizures detection by detecting seizure events from raw EEG signals. With the basis of deep neural networks and hidden Markov random field models, Fan et al. [106] proposed an unsupervised cerebrovascular segmentation method of time-of-flight magnetic resonance angiography images. Kumarasinghe et al. [107] presented a brain-driven spiking neural network framework for learning and revealing deep in time-space functional and structural patterns within spatio-temporal data.

Second, some latest studies on the detection of internalizing disorders by identifying neurobiologically informed subtypes with the basis of brain imaging data. Currently, the commonly adopted symptom-driven classification methods fail to align with underlying neurobiology. Thus, scholars are seeking alternative methods to facilitate the disorders detection. For example, Kaczkurkin et al. [108] adopted an innovative semi-supervised machine learning approach to depict patterns of neurobiological heterogeneity within adolescences with internalizing symptoms. Chen et al. [109] used a novel machine learning method for identifying a stable and generalizable factorization of the positive and negative syndrome scale and further identifying psychopathological subtypes and neurobiological differentiations.

Third, there are some latest studies focusing on the possibility of task-driven FC in individualized forecast for out-of-scanner cognitive traits. Resting and task- driven FC have been commonly adopted for characterizing human brain and cognitive abilities. Recently, scholars are seeking to extend their potentials in brain research. For example, based on large scale fMRI dataset, Jiang et al. [110] utilized machine learning methods to forecast two cognitive measures concerning reading comprehension.

Fourth, latest advances in neuroimaging and machine have significantly facilitated the exploration of cognitive processes. For example, Fincham et al. [111] proposed a hidden semi-Markov model-multi-voxel pattern-analysis approach to infer the sequence of brain states one traverses while performing cognitive tasks. Using long short-term memory recurrent neural networks, Li and Fan [112] developed an innovative framework based on deep learning for brain decoding to leverage latest progresses in intrinsic functional network modeling and sequence modeling. The proposed approach also attained encouraging decoding performance on motor and social cognition tasks.

Besides, there has been increasing interest in predicting individuals’ decision-making responses including acceptance or rejection. For example, Si et al. [113] presented an EEG-driven computational intelligence approach for predicting individuals’ responses by extracting features of discriminative spatial network pattern from single-trial brain networks with the use of a supervised learning method.

In addition, recent advances in machine learning demonstrate its potential to facilitate the judgment of different statuses of consciousness in clinical practices. For example, Campbell et al. [114] examined of machine learning algorithms trained to distinguish conscious wakefulness and anesthetic-induced unconsciousness were able to reliably identify pathologically induced unconsciousness.

## Conclusions

This study conducted a structural topic modeling based bibliometric analysis on scientific publications in AI-assisted human brain research. It explicitly reveals the prominent topics in this fast-developing, interdisciplinary field in the past decade, the different developmental trends of those topics, the diverse distributions of these topics among various types of research units, and the importance of influential research units in topical development and collaboration. It also points out several promising topics in this field. These results can induce better understanding of the latent topical popularity, dynamics, correlation, distribution, and inter-country/region collaborations in this field. They can also guide scholars and project managers to appropriately allocate resources in future research and project management practice. Moreover, by taking full advantage of the large-scale scientific data included, the proposed STM-based bibliometrics approach and analytic framework serve as a widely applicable methodological strategy to assess latent topics and development trends in an academic or practical field.

## Supporting information

S1 Fig Paper count and citation count of influential countries/regions (A), institutes (B), and journals (C) ranked by the *h*-index.
(DOCX)

S2 Fig Collaboration based on co-authorship between countries/regions with an *h*-index > = 21 (A) and institutes with an *h*-index > = 18 (B).
(DOCX)

S1 Table Final keywords list for AI research in data retrieval (search field = TS). (DOCX)

S2 Table Final keywords list for human brain research in data retrieval (search field = TS). (DOCX)

S3 Table Full names of the abbreviations (in capitals) in Table 4 in the main text. (DOCX)

S4 Table Top countries/regions in the research field, ranked by paper count in a decreasing order. (DOCX)

S5 Table Values of centrality measures for the 20 most influential countries/regions in the collaboration network. Numbers outside brackets are measure values, those within brackets are ranks of countries/regions by the corresponding measure values in a decreasing order.
(DOCX)

## References

[1] Turing A. Mind. Mind. 1950;59(236):433–60.

[2] Ullman S. Using neuroscience to develop artificial intelligence. Science. 2019;363(6428):692–3. 10.1126/science.aau6595 30765552

[3] Hassabis D, Kumaran D, Summerfield C, Botvinick M. Neuroscience-inspired artificial intelligence. Neuron. 2017;95(2):245–58. 10.1016/j.neuron.2017.06.011 28728020

[4] Arbib MA. Artificial intelligence and brain theory: unities and diversities. Annals of Biomedical Engineering. 1975;3(3):238–74. 10.1007/bf02390972 1220582

[5] Ullman S. Artificial intelligence and the brain: computational studies of the visual system. Annual Review of Neuroscience. 1986;9(1):1–26. 10.1146/annurev.ne.09.030186.000245 3518583

[6] Lee E-J, Kim Y-H, Kim N, Kang D-W. Deep into the brain: artificial intelligence in stroke imaging. Journal of Stroke. 2017;19(3):277 10.5853/jos.2017.02054 29037014 PMC5647643

[7] Shaver MM, Kohanteb PA, Chiou C, Bardis MD, Chantaduly C, Bota D, et al Optimizing Neuro-Oncology Imaging: A Review of Deep Learning Approaches for Glioma Imaging. Cancers. 2019;11(6):829. 10.3390/cancers11060829 PMC6627902 31207930

[8] Kamal H, Lopez V, Sheth SA. Machine learning in acute ischemic stroke neuroimaging. Frontiers in neurology. 2018;9:945 10.3389/fneur.2018.00945 30467491 PMC6236025

[9] Xu J, Zhang M. Use of Magnetic Resonance Imaging and Artificial Intelligence in Studies of Diagnosis of Parkinson’s Disease. ACS chemical neuroscience. 2019;10(6):2658–67. 10.1021/acschemneuro.9b00207 31083923

[10] Sakai K, Yamada K. Machine learning studies on major brain diseases: 5-year trends of 2014–2018. Japanese journal of radiology. 2019;37(1):34–72. 10.1007/s11604-018-0794-4 30498877

[11] Senders JT, Staples PC, Karhade AV, Zaki MM, Gormley WB, Broekman ML, et al Machine learning and neurosurgical outcome prediction: a systematic review. World neurosurgery. 2018;109:476–86. e1. 10.1016/j.wneu.2017.09.149 28986230

[12] Graham S, Depp C, Lee EE, Nebeker C, Tu X, Kim H-C, et al Artificial intelligence for mental health and mental illnesses: an overview. Current psychiatry reports. 2019;21(11):116 10.1007/s11920-019-1094-0 31701320 PMC7274446

[13] Aneja S, Chang E, Omuro A. Applications of artificial intelligence in neuro-oncology. Current opinion in neurology. 2019;32(6):850–6. 10.1097/WCO.0000000000000761 31609739

[14] Senders JT, Zaki MM, Karhade AV, Chang B, Gormley WB, Broekman ML, et al An introduction and overview of machine learning in neurosurgical care. Acta neurochirurgica. 2018;160(1):29–38. 10.1007/s00701-017-3385-8 29134342

[15] Chen X, Zhang X, Xie H, Wang FL, Yan J, Hao T, editors. Trends and Features of Human Brain Research Using Artificial Intelligence Techniques: A Bibliometric Approach. International Workshop on Human Brain and Artificial Intelligence; 2019: Springer.

[16] Jiang H, Qiang M, Lin P. A topic modeling based bibliometric exploration of hydropower research. Renewable and Sustainable Energy Reviews. 2016;57:226–37.

[17] Garfield E. "Science citation index"—a new dimension in indexing. Science. 1964;144(3619):649–54. 10.1126/science.144.3619.649 17806988

[18] Hassan S-U, Haddawy P, Zhu J. A bibliometric study of the world’s research activity in sustainable development and its sub-areas using scientific literature. Scientometrics. 2014;99(2):549–79.

[19] Geng Y, Chen W, Liu Z, Chiu AS, Han W, Liu Z, et al A bibliometric review: energy consumption and greenhouse gas emissions in the residential sector. Journal of Cleaner Production. 2017;159:301–16.

[20] Jiang H, Qiang M, Fan Q, Zhang M. Scientific research driven by large-scale infrastructure projects: a case study of the Three Gorges Project in China. Technological Forecasting and Social Change. 2018;134:61–71.

[21] Du HS, Ke X, Chu SK, Chan LT. A bibliometric analysis of emergency management using information systems (2000–2016). Online Information Review. 2017;41(4):454–70.

[22] Blei DM. Probabilistic topic models. Communications of the ACM. 2012;55(4):77–84.

[23] Pang J, Rao Y, Xie H, Wang X, Wang FL, Wong T-L, et al Fast Supervised Topic Models for Short Text Emotion Detection. IEEE Transactions on Cybernetics. 2019. 10.1109/TCYB.2019.2940520 31567111

[24] Yang Q, Rao Y, Xie H, Wang J, Wang FL, Chan WH, et al Segment-level joint topic-sentiment model for online review analysis. IEEE Intelligent Systems. 2019;34(1):43–50.

[25] Huang M, Rao Y, Liu Y, Xie H, Wang FL, editors. Siamese network-based supervised topic modeling. Proceedings of the 2018 Conference on Empirical Methods in Natural Language Processing; 2018.

[26] Rao Y, Xie H, Li J, Jin F, Wang FL, Li Q. Social emotion classification of short text via topic-level maximum entropy model. Information & Management. 2016;53(8):978–86.

[27] Roberts ME, Stewart BM, Tingley D. stm: R package for structural topic models. Journal of Statistical Software. 2014;10(2):1–40.

[28] Roberts ME, Stewart BM, Tingley D, Lucas C, Leder‐Luis J, Gadarian SK, et al Structural topic models for open‐ended survey responses. American Journal of Political Science. 2014;58(4):1064–82.

[29] Bagozzi BE, Berliner D. The politics of scrutiny in human rights monitoring: evidence from structural topic models of US State Department human rights reports. Political Science Research and Methods. 2018;6(4):661–77.

[30] Chen X, Xie H, Wang FL, Liu Z, Xu J, Hao T. A bibliometric analysis of natural language processing in medical research. BMC Medical Informatics and Decision Making. 2018;18(1):14. 29589569 10.1186/s12911-018-0594-x PMC5872501

[31] Farrell J. Corporate funding and ideological polarization about climate change. Proceedings of the National Academy of Sciences of the USA. 2016;113(1):92–7. 10.1073/pnas.1509433112 26598653 PMC4711825

[32] Mann HB. Nonparametric tests against trend. Econometrica: Journal of the Econometric Society. 1945;13:245–59.

[33] Moed H, De Bruin R, Van Leeuwen T. New bibliometric tools for the assessment of national research performance: database description, overview of indicators and first applications. Scientometrics. 1995;33(3):381–422.

[34] Zhu Q, Kong X, Hong S, Li J, He Z. Global ontology research progress: a bibliometric analysis. Aslib Journal of Information Management. 2015;67(1):27–54.

[35] Hao T, Chen X, Li G, Yan J. A bibliometric analysis of text mining in medical research. Soft Computing. 2018;22(23):7875–92.

[36] Song Y, Chen X, Hao T, Liu Z, Lan Z. Exploring two decades of research on classroom dialogue by using bibliometric analysis. Computers & Education. 2019;137:12–31.

[37] Chen X, Zou D, Cheng G, Xie H. Detecting latent topics and trends in educational technologies over four decades using structural topic modeling: A retrospective of all volumes of computer & education. Computers & Education. 2020:103855 10.1016/j.compedu.2020.103855.

[38] Chen X, Zou D, Xie H. Fifty years of British Journal of Educational Technology: A topic modeling based bibliometric perspective. British Journal of Educational Technology. n/a(n/a). 10.1111/bjet.12907

[39] Chen X, Yu G, Cheng G, Hao T. Research topics, author profiles, and collaboration networks in the top-ranked journal on educational technology over the past 40 years: a bibliometric analysis. Journal of Computers in Education. 2019:1–23.

[40] Chen X, Wang S, Tang Y, Hao T. A bibliometric analysis of event detection in social media. Online Information Review. 2019;43(1):29–52.

[41] Chen X, Liu Z, Wei L, Yan J, Hao T, Ding R. A comparative quantitative study of utilizing artificial intelligence on electronic health records in the USA and China during 2008–2017. BMC Medical Informatics and Decision Making. 2018;18(5):117. 30526643 10.1186/s12911-018-0692-9 PMC6284279

[42] Chen X, Lun Y, Yan J, Hao T, Weng H. Discovering thematic change and evolution of utilizing social media for healthcare research. BMC Medical Informatics and Decision Making. 2019;19(2):50. 30961624 10.1186/s12911-019-0757-4 PMC6454597

[43] Chen X, Ding R, Xu K, Wang S, Hao T, Zhou Y. A bibliometric review of natural language processing empowered mobile computing. Wireless Communications and Mobile Computing. 2018;2018:1827074.

[44] Alonso S, Cabrerizo F, Herrera-Viedma E, Herrera F. hg-index: A new index to characterize the scientific output of researchers based on the h-and g-indices. Scientometrics. 2009;82(2):391–400.

[45] Gutiérrez-Salcedo M, Martínez MÁ, Moral-Munoz J, Herrera-Viedma E, Cobo MJ. Some bibliometric procedures for analyzing and evaluating research fields. Applied Intelligence. 2018:1–13.

[46] Gimenez E, Salinas M, Manzano-Agugliaro F. Worldwide research on plant defense against biotic stresses as improvement for sustainable agriculture. Sustainability. 2018;10(2):391.

[47] Roberts ME, Stewart BM, Airoldi EM. A model of text for experimentation in the social sciences. Journal of the American Statistical Association. 2016;111(515):988–1003.

[48] Forsythe GE, Malcolm MA, Moler CB. Computer methods for mathematical computations: Prentice-hall Englewood Cliffs, NJ; 1977.

[49] Zhao T, Liu H, Roeder K, Lafferty J, Wasserman L. The huge package for high-dimensional undirected graph estimation in R. Journal of Machine Learning Research. 2012;13(Apr):1059–62. 26834510 PMC4729207

[50] Hoekzema E, Schagen SE, Kreukels BP, Veltman DJ, Cohen-Kettenis PT, Delemarre-van de Waal H, et al Regional volumes and spatial volumetric distribution of gray matter in the gender dysphoric brain. Psychoneuroendocrinology. 2015;55:59–71. 10.1016/j.psyneuen.2015.01.016 25720349

[51] Geytenbeek JJ, Vermeulen RJ, Becher JG, Oostrom KJ. Comprehension of spoken language in non‐speaking children with severe cerebral palsy: an explorative study on associations with motor type and disabilities. Developmental Medicine & Child Neurology. 2015;57(3):294–300. 25349105 10.1111/dmcn.12619

[52] Siuly S, Li Y. A novel statistical algorithm for multiclass EEG signal classification. Engineering Applications of Artificial Intelligence. 2014;34:154–67.

[53] Yang R, Huang HM, Hong QH, Yin XB, Tan ZH, Shi T, et al Synaptic suppression triplet‐STDP learning rule realized in second‐order memristors. Advanced Functional Materials. 2018;28(5):1704455.

[54] Lancione M, Tosetti M, Donatelli G, Cosottini M, Costagli M. The impact of white matter fiber orientation in single‐acquisition quantitative susceptibility mapping. NMR in Biomedicine. 2017;30(11):e3798. 10.1002/nbm.3798 28902421

[55] Zhou X, Jiang X, Ye Z, Zhang Y, Lou K, Zhan W. Semantic integration processes at different levels of syntactic hierarchy during sentence comprehension: an ERP study. Neuropsychologia. 2010;48(6):1551–62. 10.1016/j.neuropsychologia.2010.02.001 20138898

[56] Yang J, Lu L, Tan W, Song Y, Yan J, Deng M, et al A Modified MRF Algorithm Based on Neighborhood Spatial Information for MRI Brain Tissue Segmentation. Journal of Medical Imaging and Health Informatics. 2017;7(7):1525–30.

[57] Park J, Bucher ES, Fontillas K, Owesson-White C, Ariansen JL, Carelli RM, et al Opposing catecholamine changes in the bed nucleus of the stria terminalis during intracranial self-stimulation and its extinction. Biological psychiatry. 2013;74(1):69–76. 10.1016/j.biopsych.2012.11.008 23260335 PMC3609919

[58] Soch J, Allefeld C. MACS–a new SPM toolbox for model assessment, comparison and selection. Journal of neuroscience methods. 2018;306:19–31. 10.1016/j.jneumeth.2018.05.017 29842901

[59] Parsopoulos KE, Kariotou F, Dassios G, Vrahatis MN. Tackling magnetoencephalography with particle swarm optimization. International Journal of Bio-Inspired Computation. 2009;1(1–2):32–49.

[60] Jeong W, Chung CK, Kim JS. Magnetoencephalography interictal spike clustering in relation with surgical outcome of cortical dysplasia. Journal of Korean Neurosurgical Society. 2012;52(5):466 10.3340/jkns.2012.52.5.466 23323167 PMC3539081

[61] Kathirvel R, Batri K. A computer‐aided approach for meningioma brain tumor detection using C ANFIS classifier. International Journal of Imaging Systems and Technology. 2017;27(3):193–200.

[62] Li X, Cui L, Tao S, Chen J, Zhang X, Zhang G-Q. Hyclasss: a hybrid classifier for automatic sleep stage scoring. IEEE journal of biomedical and health informatics. 2017;22(2):375–85. 10.1109/JBHI.2017.2668993 28222004

[63] Stalmans S, Gevaert B, Wynendaele E, Nielandt J, De Tré G, Peremans K, et al Classification of peptides according to their blood-brain barrier influx. Protein and Peptide Letters. 2015;22(9):768–75. 10.2174/0929866522666150622101223 26095378

[64] Schettini F, Aloise F, Aricò P, Salinari S, Mattia D, Cincotti F. Self-calibration algorithm in an asynchronous P300-based brain–computer interface. Journal of neural engineering. 2014;11(3):035004 10.1088/1741-2560/11/3/035004 24838347

[65] Kraus D, Naros G, Bauer R, Leão MT, Ziemann U, Gharabaghi A. Brain–robot interface driven plasticity: distributed modulation of corticospinal excitability. Neuroimage. 2016;125:522–32. 10.1016/j.neuroimage.2015.09.074 26505298

[66] Yu P, Dean RA, Hall SD, Qi Y, Sethuraman G, Willis BA, et al Enriching amnestic mild cognitive impairment populations for clinical trials: optimal combination of biomarkers to predict conversion to dementia. Journal of Alzheimer's disease. 2012;32(2):373–85. 10.3233/JAD-2012-120832 22796873

[67] Wang R, Zhang Z-Z, Ma J, Yang Y, Lin P, Wu Y. Spectral properties of the temporal evolution of brain network structure. Chaos. 2015;25(12):123112 10.1063/1.4937451 26723151

[68] Deen B, Pitskel NB, Pelphrey KA. Three systems of insular functional connectivity identified with cluster analysis. Cerebral cortex. 2011;21(7):1498–506. 10.1093/cercor/bhq186 21097516 PMC3116731

[69] Blüml S, Margol AS, Sposto R, Kennedy RJ, Robison NJ, Vali M, et al Molecular subgroups of medulloblastoma identification using noninvasive magnetic resonance spectroscopy. Neuro-oncology. 2015;18(1):126–31. 10.1093/neuonc/nov097 26254476 PMC4677409

[70] Mourik JE, Lubberink M, Van Velden FH, Kloet RW, Van Berckel BN, Lammertsma AA, et al In vivo validation of reconstruction-based resolution recovery for human brain studies. Journal of Cerebral Blood Flow & Metabolism. 2010;30(2):381–9. 19844240 10.1038/jcbfm.2009.225 PMC2949117

[71] Orlov T, Zohary E. Object representations in human visual cortex formed through temporal integration of dynamic partial shape views. Journal of Neuroscience. 2018;38(3):659–78. 10.1523/JNEUROSCI.1318-17.2017 29196319 PMC6596194

[72] Petrantonakis PC, Hadjileontiadis LJ. A novel emotion elicitation index using frontal brain asymmetry for enhanced EEG-based emotion recognition. IEEE Transactions on Information Technology in Biomedicine. 2011;15(5):737–46. 10.1109/TITB.2011.2157933 21622077

[73] Goto Y, Maeda T, Nakatsu-Goto Y. Decision tree model for predicting long-term outcomes in children with out-of-hospital cardiac arrest: a nationwide, population-based observational study. Critical Care. 2014;18(3):R133 10.1186/cc13951 24972847 PMC4227055

[74] Hiar RE, Haddad S, Jaïdane H, Hober D, M’hadheb-Gharbi MB, Gullberg M, et al Enteroviral central nervous system infections in children of the region of monastir, Tunisia: diagnosis, laboratory findings of cerebrospinal fluid and clinical manifestations. Indian Journal of Virology. 2012;23(3):294–302. 10.1007/s13337-012-0104-1 24293816 PMC3550789

[75] Bartsch MV, Donohue SE, Strumpf H, Schoenfeld MA, Hopf J-M. Enhanced spatial focusing increases feature-based selection in unattended locations. Scientific Reports. 2018;8(1):16132 10.1038/s41598-018-34424-5 30382137 PMC6208401

[76] Perez-Magan E, Rodríguez de Lope Á, Ribalta T, Ruano Y, Campos-Martín Y, Perez-Bautista G, et al Differential expression profiling analyses identifies downregulation of 1p, 6q, and 14q genes and overexpression of 6p histone cluster 1 genes as markers of recurrence in meningiomas. Neuro-Oncology. 2010;12(12):1278–90. 10.1093/neuonc/noq081 20685720 PMC3018937

[77] Guo W, Su Q, Yao D, Jiang J, Zhang J, Zhang Z, et al Decreased regional activity of default-mode network in unaffected siblings of schizophrenia patients at rest. European Neuropsychopharmacology. 2014;24(4):545–52. 10.1016/j.euroneuro.2014.01.004 24491950

[78] Li G, Chung W-Y. A context-aware EEG headset system for early detection of driver drowsiness. Sensors. 2015;15(8):20873–93. 10.3390/s150820873 26308002 PMC4570452

[79] Hernandez-Meza G, Izzetoglu M, Osbakken M, Green M, Abubakar H, Izzetoglu K. Investigation of optical neuro-monitoring technique for detection of maintenance and emergence states during general anesthesia. Journal of Clinical Monitoring and Computing. 2018;32(1):147–63. 10.1007/s10877-017-9998-x 28214930

[80] Iakovidou ND, Dimitriadis SI, Laskaris NA, Tsichlas K, Manolopoulos Y. On the discovery of group-consistent graph substructure patterns from brain networks. Journal of Neuroscience Methods. 2013;213(2):204–13. 10.1016/j.jneumeth.2012.12.018 23274947

[81] Guo L, Wu Y, Zhao L, Cao T, Yan W, Shen X. Classification of mental task from EEG signals using immune feature weighted support vector machines. IEEE Transactions on Magnetics. 2010;47(5):866–9.

[82] Sacchet MD, Prasad G, Foland-Ross LC, Thompson PM, Gotlib IH. Support vector machine classification of major depressive disorder using diffusion-weighted neuroimaging and graph theory. Frontiers in Psychiatry. 2015;6:21 10.3389/fpsyt.2015.00021 25762941 PMC4332161

[83] Oweis RJ, Abdulhay EW. Seizure classification in EEG signals utilizing Hilbert-Huang transform. Biomedical Engineering Online. 2011;10(1):38. 21609459 10.1186/1475-925X-10-38 PMC3116477

[84] Alçіn ÖF, Siuly S, Bajaj V, Guo Y, Şengu A, Zhang Y. Multi-category EEG signal classification developing time-frequency texture features based Fisher Vector encoding method. Neurocomputing. 2016;218:251–8.

[85] Huang M, Xie H, Rao Y, Feng J, Wang FL. Sentiment Strength Detection With a Context-dependent Lexicon-based Convolutional Neural Network. Information Sciences. 2020.

[86] Li X, Rao Y, Xie H, Liu X, Wong T-L, Wang FL. Social emotion classification based on noise-aware training. Data & Knowledge Engineering. 2019;123:101605.

[87] Liang W, Xie H, Rao Y, Lau RY, Wang FL. Universal affective model for Readers’ emotion classification over short texts. Expert Systems with Applications. 2018;114:322–33.

[88] Li X, Rao Y, Xie H, Lau RYK, Yin J, Wang FL. Bootstrapping social emotion classification with semantically rich hybrid neural networks. IEEE Transactions on Affective Computing. 2017;8(4):428–42.

[89] Jie B, Wee CY, Shen D, Zhang D. Hyper-connectivity of functional networks for brain disease diagnosis. Medical Image Analysis. 2016;32:84–100. 10.1016/j.media.2016.03.003 27060621 PMC5333488

[90] Brown JA, Rudie JD, Bandrowski A, Van Horn JD, Bookheimer SY. The UCLA multimodal connectivity database: a web-based platform for brain connectivity matrix sharing and analysis. Frontiers in Neuroinformatics. 2012;6:28 10.3389/fninf.2012.00028 23226127 PMC3508475

[91] Bassett DS, Zurn P, Gold JI. On the nature and use of models in network neuroscience. Nature Reviews Neuroscience. 2018;19(9):566–78. 10.1038/s41583-018-0038-8 30002509 PMC6466618

[92] Yamins DL, DiCarlo JJ. Using goal-driven deep learning models to understand sensory cortex. Nature Neuroscience. 2016;19(3):356 10.1038/nn.4244 26906502

[93] Hubel DH, Wiesel TN. Receptive fields of single neurones in the cat's striate cortex. Journal of Physiology. 1959;148(3):574–91. 14403679 10.1113/jphysiol.1959.sp006308 PMC1363130

[94] Orhan U, Hekim M, Ozer M. EEG signals classification using the k-means clustering and a multilayer perceptron neural network model. Expert Systems with Applications. 2011;38(10):13475–81.

[95] Nicolaou N, Georgiou J. Neural network–based classification of anesthesia/awareness using granger causality features. Clinical EEG and Neuroscience. 2014;45(2):77–88. 10.1177/1550059413486271 23820086

[96] Nieminen J. On the centrality in a graph. Scandinavian journal of psychology. 1974;15(1):332–6. 4453827 10.1111/j.1467-9450.1974.tb00598.x

[97] Bavelas A. A mathematical model for group structures. Human organization. 1948;7(3):16.

[98] Newman ME. A measure of betweenness centrality based on random walks. Social networks. 2005;27(1):39–54.

[99] Bonacich P. Technique for analyzing overlapping memberships. Sociological methodology. 1972;4:176–85.

[100] Thagard P. Theory and experiment in cognitive science. Artificial Intelligence. 2007;171(18):1104–6.

[101] Van Gulick R. Higher-order global states (HOGS): an alternative higher-order model. Higher-Order Theories of Consciousness. 2004:67–93.

[102] Raichle ME, MacLeod AM, Snyder AZ, Powers WJ, Gusnard DA, Shulman GL. A default mode of brain function. Proceedings of the National Academy of Sciences of the USA. 2001;98(2):676–82. 10.1073/pnas.98.2.676 11209064 PMC14647

[103] Hong T, Shuai L, Frost SJ, Landi N, Pugh KR, Shu H. Cortical responses to Chinese phonemes in preschoolers predict their literacy skills at school age. Developmental Neuropsychology. 2018;43(4):356–69. 10.1080/87565641.2018.1439946 29521532 PMC5986580

[104] Chen L, Gong T, Kosinski M, Stillwell D, Davidson RL. Building a profile of subjective well-being for social media users. PLoS One. 2017;12(11):e0187278 10.1371/journal.pone.0187278 29135991 PMC5685571

[105] O’Shea A, Lightbody G, Boylan G, Temko A. Neonatal seizure detection from raw multi-channel EEG using a fully convolutional architecture. Neural Networks. 2020;123:12–25. 10.1016/j.neunet.2019.11.023 31821947

[106] Fan S, Bian Y, Chen H, Kang Y, Yang Q, Tan T. Unsupervised Cerebrovascular Segmentation of TOF-MRA Images Based on Deep Neural Network and Hidden Markov Random Field Model. Frontiers in Neuroinformatics. 2020;13:77 10.3389/fninf.2019.00077 31998107 PMC6965699

[107] Kumarasinghe K, Kasabov N, Taylor D. Deep learning and deep knowledge representation in Spiking Neural Networks for Brain-Computer Interfaces. Neural Networks. 2020;121:169–85. 10.1016/j.neunet.2019.08.029 31568895

[108] Kaczkurkin AN, Sotiras A, Baller EB, Barzilay R, Calkins ME, Chand GB, et al Neurostructural heterogeneity in youths with internalizing symptoms. Biological psychiatry. 2020;87(5):473–82. 10.1016/j.biopsych.2019.09.005 31690494 PMC7007843

[109] Chen J, Patil KR, Weis S, Sim K, Nickl-Jockschat T, Zhou J, et al Neurobiological Divergence of the Positive and Negative Schizophrenia Subtypes Identified on a New Factor Structure of Psychopathology Using Non-negative Factorization: An International Machine Learning Study. Biological psychiatry. 2020;87(3):282–93. 10.1016/j.biopsych.2019.08.031 31748126 PMC6946875

[110] Jiang R, Zuo N, Ford JM, Qi S, Zhi D, Zhuo C, et al Task-induced brain connectivity promotes the detection of individual differences in brain-behavior relationships. NeuroImage. 2020;207:116370 10.1016/j.neuroimage.2019.116370 31751666 PMC7345498

[111] Fincham JM, Lee HS, Anderson JR. Spatiotemporal analysis of event‐related fMRI to reveal cognitive states. Human brain mapping. 2020;41(3):666–83. 10.1002/hbm.24831 31725183 PMC7267968

[112] Li H, Fan Y. Interpretable, highly accurate brain decoding of subtly distinct brain states from functional MRI using intrinsic functional networks and long short-term memory recurrent neural networks. NeuroImage. 2019;202:116059 10.1016/j.neuroimage.2019.116059 31362049 PMC6819260

[113] Si Y, Li F, Duan K, Tao Q, Li C, Cao Z, et al Predicting individual decision-making responses based on single-trial EEG. NeuroImage. 2020;206:116333. 10.1016/j.neuroimage.2019.116333 31698078

[114] Campbell JM, Huang Z, Zhang J, Wu X, Qin P, Northoff G, et al Pharmacologically informed machine learning approach for identifying pathological states of unconsciousness via resting-state fMRI. NeuroImage. 2020;206:116316 10.1016/j.neuroimage.2019.116316 31672663 PMC6981054

