# Supplementary material for: Topics and trends in artificial intelligence assisted human brain research
Source: PLoS One. 2020 Apr 6;15(4):e0231192. doi: 10.1371/journal.pone.0231192 (PMC7135272; doi:10.1371/journal.pone.0231192)
Supplement: S1 Fig — Paper count and citation count of influential countries/regions (A), institutes (B), and journals (C) ranked by the h-index. (DOCX) [file pone.0231192.s001.docx]

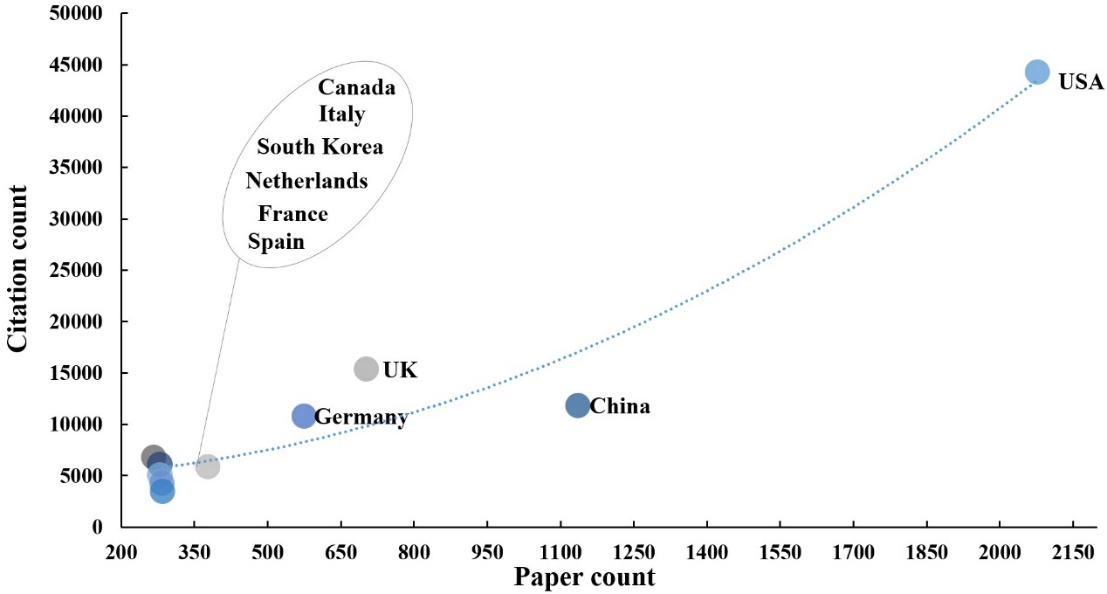


(A)


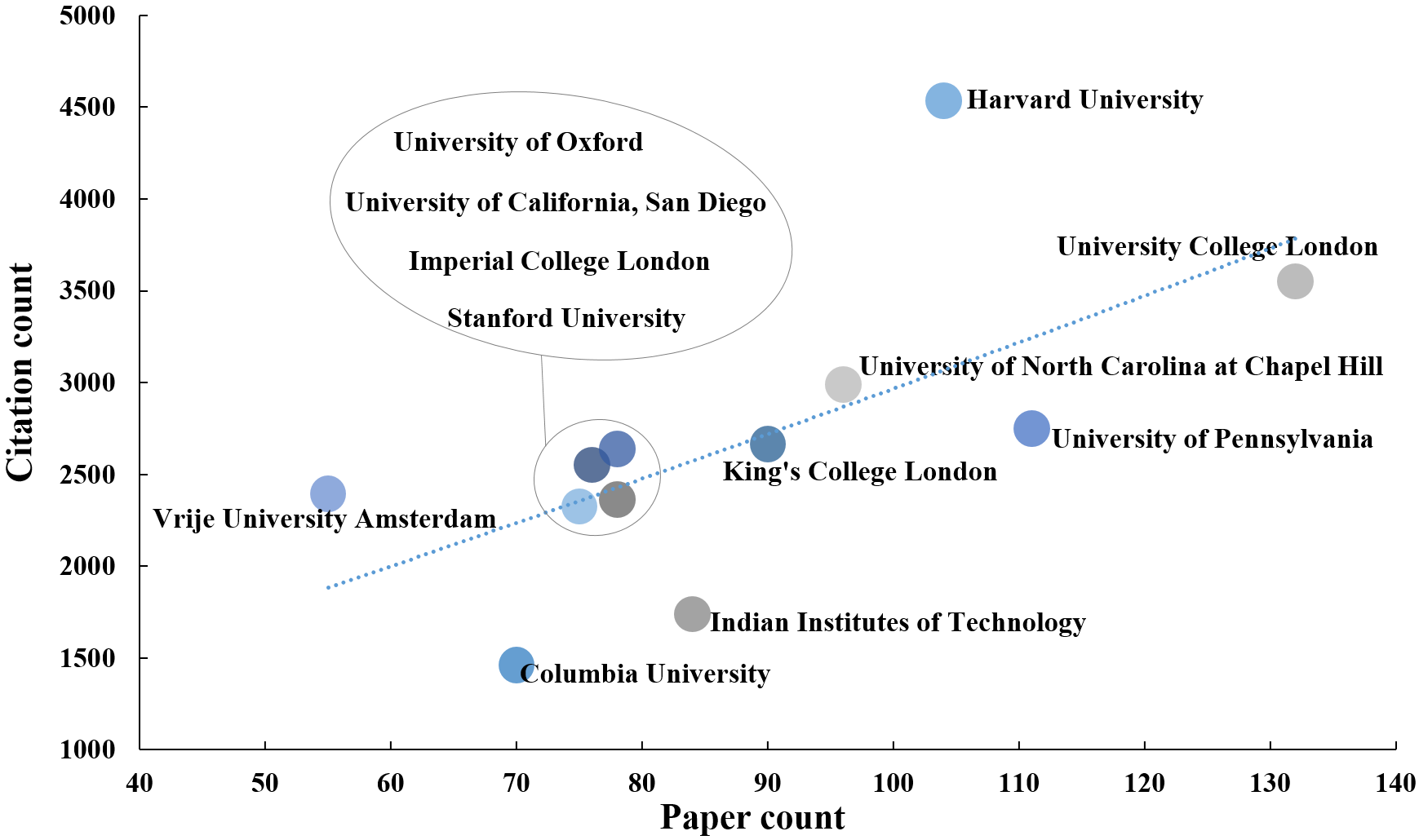


(B)

**
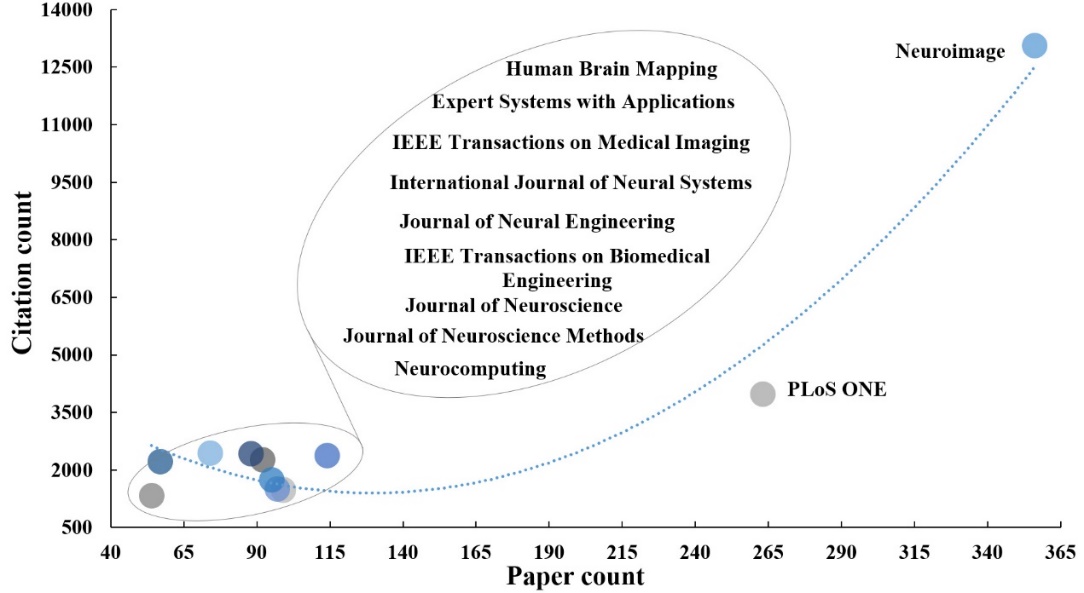
**

(C)

**S1 Fig. Paper count and citation count of influential countries/regions (A), institutes (B), and journals (C) ranked by the *h*-index.**
