# Supplementary material for: Topics and trends in artificial intelligence assisted human brain research
Source: PLoS One. 2020 Apr 6;15(4):e0231192. doi: 10.1371/journal.pone.0231192 (PMC7135272; doi:10.1371/journal.pone.0231192)
Supplement: S2 Fig — Collaboration based on co-authorship between countries/regions with an h-index > = 21 (A) and institutes with an h-index > = 18 (B). (DOCX) [file pone.0231192.s002.docx]

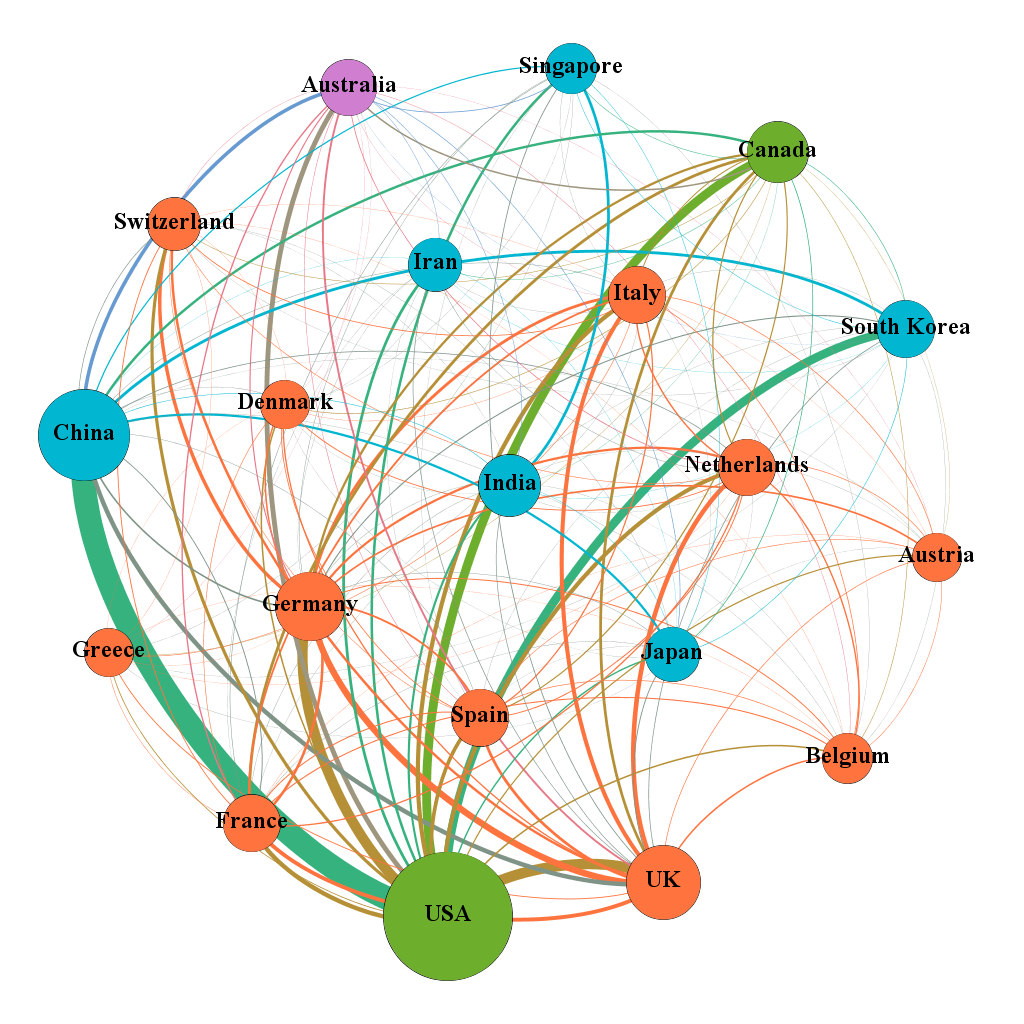


(A)


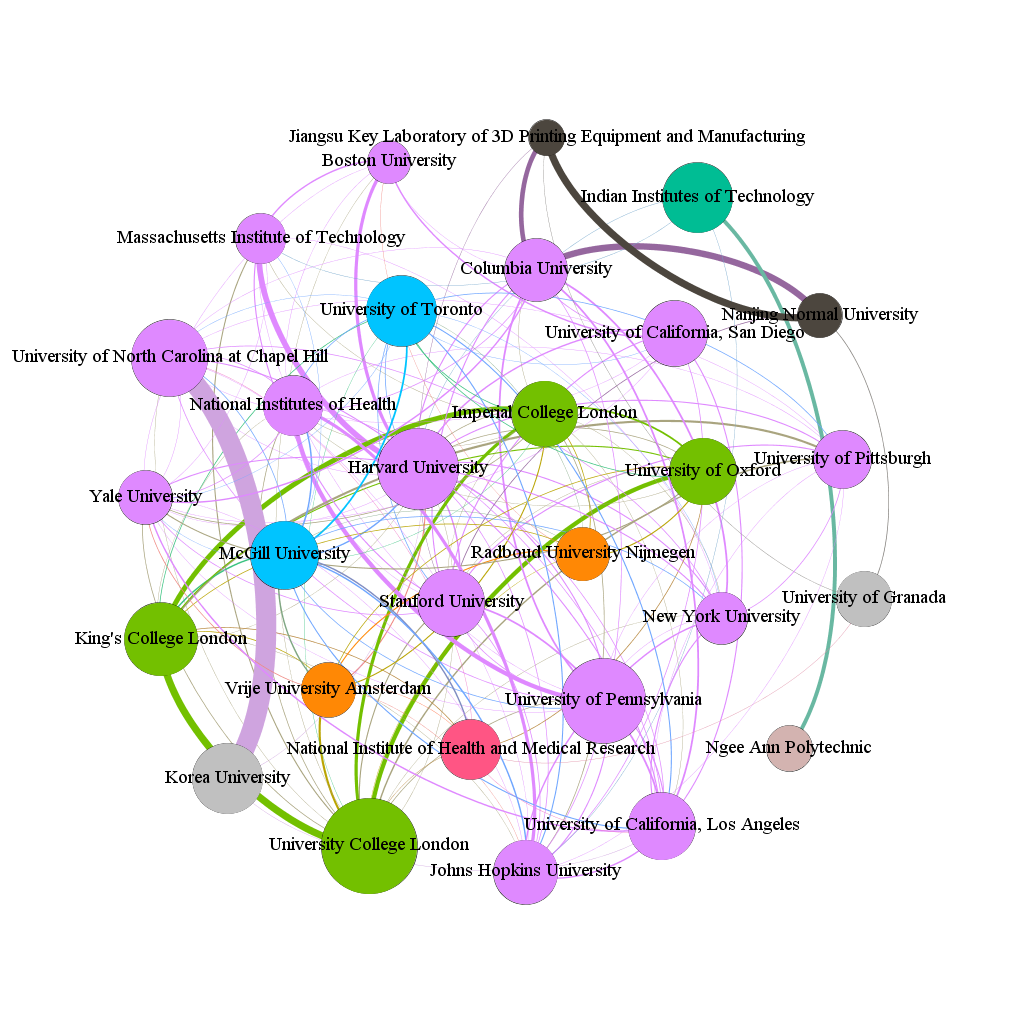


(B)

**S2 Fig. Collaboration based on co-authorship between countries/regions with an *h*-index >=21 (A) and institutes with an *h*-index >=18 (B).**
