# Supplementary material for: Topics and trends in artificial intelligence assisted human brain research
Source: PLoS One. 2020 Apr 6;15(4):e0231192. doi: 10.1371/journal.pone.0231192 (PMC7135272; doi:10.1371/journal.pone.0231192)
Supplement: S1 Table — (DOCX) [file pone.0231192.s003.docx]

**S1 Table. Final keywords list for AI research in data retrieval (search field=TS).**

| "vision understanding” or “scene understanding” or “semantic technology” or “k nearest neighbor” or “classifier” or “cnn feature” or “data fusion” or “data mining” or “fuzzy logic” or “neuro-fuzzy” or “text mining” or “text-mining” or “deep network” or “fuzzy system” or “graph mining” or “image fusion” or “markov chain” or “neural fuzzy” or “neural-fuzzy” or “semantic web” or “bayes network” or “decision tree” or “deep learning” or “deep-learning” or “fusion method” or “fuzzy control” or “karnik-mendel” or “kernel method” or “kinetic model” or “random forest” or “visual search” or “bayes learning” or “bayesian brain” or “cfs clustering” or “cloud robotics” or “face alignment” or “face detection” or “face synthesis” or “feature coding” or “feature fusion” or “fundus imaging” or “gmcc algorithm” or “hybrid feature” or “image analysis” or “image forensic” or “multiclass svm” or “naive bayesian” or “neural control” or “neural network” or “random feature” or “random network” or “robust control” or “smart learning” or “structural svm” or “cluster finding” or “computer vision” or “data clustering” or “face clustering” or “features mining” or “fusion decision” or “fuzzy reasoning” or “graph embedding” or “hybrid coupling” or “knowledge graph” or “learning kernel” or “learning system” or “motion planning” or “neural learning” or “nonparallel svm” or “regression tree” or “retinal imaging” or “text clustering” or “bayesian network” or “cloud automation” or “cluster analysis” or “face recognition” or “feature learning” or “fuzzy clustering” or “fuzzy set theory” or “greedy algorithm” or “hashing learning” or “hopfield network” or “hybrid microgrid” or “image annotation” or “learning to hash” or “machine learning” or “machine-learning” or “naive bayes tree” or “nearest neighbor” or “nearest-neighbor” or “pattern analysis” or “rough set theory” or “semantic mapping” or “text recognition” or “adaptive learning” or “big data learning” or “clustering method” or “ensemble learning” or “firefly algorithm” or “genetic algorithm” or “implicit learning” or “intelligent agent” or “k-means algorithm” or “language modeling” or “manifold learning” or “multiplex network” or “nearest neighbour” or “neural nets model” or “ontology matching” or “pattern discovery” or “pattern selection” or “place recognition” or “scene recognition” or “semantic analysis” or “semantic labeling” or “semantic saliency” or “sequence analysis” or “sequence learning” or “transfer learning” or “virtual assistant” or “action recognition” or “bayesian inference” or “cnn implementation” or “discriminative cnn” or “facial recognition” or “granular computing” or “hesitant fuzzy set” or “human intelligence” or “image segmentation” or “intelligent system” or “k-means clustering” or “k-nearest neigbour” or “machine perception” or “object recognition” or “ontology alignment” or “question answering” or “scattering network” or “sentiment analysis” or “speech recognition” or “supervised hashing” or “swarm intelligence” or “affective computing” or “appearance modeling” or “automated inference” or “automated reasoning” or “classification tree” or “clustering analysis” or “deep belief network” or “dictionary learning” or “emotion recognition” or “fuzzy cognitive map” or “image understanding” or “implicit cognition” or “intelligent control” or “knowledge discovery” or “machine translation” or “markov random field” or “nearly-isotonic svm” or “pattern recognition” or “supervised learning” or “text classification” or “variational network” or “ambient intelligence” or “brain-based learning” or “clustering algorithm” or “human-machine system” or “image categorization” or “image classification” or “image reconstruction” or “image-based modeling” or “intelligent approach” or “knowledge extraction” or “machine intelligence” or “multi-modal learning” or “pixel classification” or “predictive analytics” or “scene classification” or “semantic orientation” or “tensor-train network” or “binary classification” or “business intelligence” or “information retrieval” or “intelligent computing” or “knowledge-based model” or “man-machine interface” or “semantic segmentation” or “semantic technologies” or “visual classification” or “cross-view recognition” or “emotion classification” or “expression recognition” or “feature representation” or “gaussian mixture model” or “image change detection” or “information extraction” or “intuitive intelligence” or “language comprehension” or “partitional clustering” or “pattern classification” or “reinforcement learning” or “support vector machine” or “support vector network” or “technical intelligence” or “actionable intelligence” or “activity classification” or “analytical intelligence” or “artificial intelligence” or “association rule mining” or “bayesian belief network” or “brain-machine interface” or “classification analysis” or “cluster synchronization” or “competitor intelligence” or “decision support system” or “deep polynomial network” or “empathetic intelligence” or “ensemble classification” or “filter weights learning” or “handwriting recognition” or “hierarchical clustering” or “intelligent computation” or “mechanical intelligence” or “monte carlo tree search” or “object detection” or “neighborhood similarity” or “particle identification” or “proximal classification” or “representation learning” or “semantic topic analysis” or “semi-auto image tagging” or “support vectors machine” or “unsupervised clustering” or “visual word recognition” or “association link network” or “classification algorithm” or “class-imbalance learning” or “competitive intelligence” or “conditional random field” or “evolutionary computation” or “learning machine” or “fuzzy c-means clustering” or “activity detection” or “image set classification” or “intelligent robot system” or “multiple kernel learning” or “multiple-kernel-learning” or “named entity recognition” or “natural image statistics” or “natural scene statistics” or “networked control system” or “person re-identification” or “sentiment classification” or “bayesian learning” or “features learning” or “switched network cluster” or “artificial neural-network” or “causal structure learning” or “ecg signal classification” or “gradient boosting machine” or “imbalanced classification” or “opposition-based learning” or “supervised classification” or “trajectory classification” or “pattern mining” or “word sense disambiguation” or “composite learning control” or “computational intelligence” or “deep contour-aware network” or “feature detection” or “large-scale classification” or “learning-based synchronous” or “multi-label classification” or “multiple-instance learning” or “naive bayes classification” or “neural dynamic programming” or “online sequential learning” or “perspectives on clustering” or “action/activity recognition” or “fuzzy classification” or “gustafson-kessel clustering” or “multilingual knowledge base” or “natural language generation” or “natural language processing” or “automatic target recognition” or “face and gesture recognition” or “interactive visual analytics” or “structure learning algorithm” or “content-based image retrieval” or “handwritten digit recognition” or “feature selection” or “negative correlation learning” or “support vector classification” or “intelligent learning” or “fuzzy knowledge representation” or “natural language understanding” or “artificial bee colony algorithm” or “artificial fish swarm algorithm” or “artificial general intelligence” or “contrastive divergence learning” or “nearest subspace classification” or “nonnegative spectral clustering” or “spectral-spatial classification” or “target tracking” or “highly imbalanced classification” or “excavation equipments recognition” or “sound classification” or “intelligent information processing” or “imagery classification” or “laplacian sparse subspace clustering” or “novel intelligent damping controller” or “classification method” or “fuzzy inference” or “connectionist temporal classification" |
| --- |
