# Supplementary material for: Topics and trends in artificial intelligence assisted human brain research
Source: PLoS One. 2020 Apr 6;15(4):e0231192. doi: 10.1371/journal.pone.0231192 (PMC7135272; doi:10.1371/journal.pone.0231192)
Supplement: S2 Table — (DOCX) [file pone.0231192.s004.docx]

**S2 Table. Final keywords list for human brain research in data retrieval (search field=TS).**

| Case 1 | "brainnetome” or “brain mapping” or “brain development” or “brain aging” or “brain health” or “brain disease” or “brain rehabilitation” or “brain death” or “brain disorder” or “electroencephalogram” or “functional magnetic resonance imaging” or “deep brain stimulation” or “magnetoencephalography” or “brain intelligence” or “transcranial magnetic stimulation” or “brain connectivity” or “fmri” or “fcmri” or “drebrinbrainmap” or “cerebrum” or “dopamine” or “thalamus” or “brainstem” or “gut-brain” or “meningioma” or “meningitis” or “brain injury” or “brain trauma” or “encephalitis” or “angular gyrus” or “brain atrophy” or “cerebral vein” or “mirror neuron” or “ventriculitis” or “brain activity” or “brain injuries” or “brain ischemia” or “brain necrosis” or “brain response” or “brain training” or “brain-gut axis” or “encephalopathy” or “brain evolution” or “brain magnetite” or “brain targeting” or “cerebrovascular” or “locus coeruleus” or “medulloblastoma” or “brain metastases” or “brain metastasis” or “brain monoaminesbrain amyloidosis” or “brain asymmetries” or “brain parcelation” or “cerebral arteries” or “cerebral ischemia” or “encephalomyelitis” or “epileptic seizure” or “hyperexcitability” or “post-mortem brain” or “psychotic symptom” or “brain segmentation” or “adult neurogenesis” or “affective disorder” or “anterior cingulate” or “braak tangle stage” or “cerebral ischaemia” or “depressive symptom” or “divergent thinking” or “eeg classification” or “endocannabinoidome” or “fluid intelligence” or “incentive salience” or “inhibitory control” or “lateral ventricles” or “anesthetic toxicity” or “blood-brain barrier” or “brain-gd deposition” or “cerebellar function” or “cerebral infarction” or “cerebral microbleed” or “cerebral-protection” or “cerebrospinal fluid” or “cholinergic neurons” or “eeg signal analysis” or “lacunar infarctions” or “neurologic disorder” or “psychosocial factor” or “sensory attenuation” or “subthalamic nucleus” or “action understanding” or “brain hypometabolism” or “cerebral circulation” or “docosahexaenoic acid” or “encephalogram signal” or “posteromedial cortex" |
| --- | --- |
| OR | |
| Case 2 | "brain” or “multi-voxel pattern analysis” or “diffusion tensor imaging” or “functional connectivity” or “dynamic casual modeling” or “positron emission tomography” or “cerebral” or “insula” or “pet/ct” or “eeg-tms” or “fdg pet” or “fdg-pet” or “pet/mri” or “pet-fdg” or “pet imaging” or “eeg analysis” or “eeg artefact” or “eeg artifact” or “invasive eeg” or “diffusion mri” or “functional mri” or “focal eeg signal” or “interhemispheric” or “obsessive-compulsive disorder” or “dorsolateral prefrontal cortex” or “gaba-acting antiepileptic drug” or “sentence comprehension deficit” or “effective transverse relaxation” or “intracerebral dipole generators” or “psychophysiological interaction” or “sudden sensorineural hearing loss” or “hypothalamo-pituitary-adrenal axis” or “hypothalamic pituitary adrenal axis” or “hypothalamic-pituitary-adrenal axis” or “quantitative susceptibility mapping” or “cholinergic anti-inflammatory pathway” or “functional magnetic-resonance imaging” or “resting state functional connectivity” or “resting-state functional connectivity” or “executive functions and speed of processing" |
|  | AND |
|  | "perception” or “imagery” or “vision” or “audition” or “somatic sensory” or “movement” or “motivation” or “emotion” or “rhythm” or “sleep” or “language” or “attention” or “learning” or “memory” or “disease” or “neuroimaging” or “neuroinformatics” or “mood” or “fear” or “mania” or “apathy” or “autism” or “stress” or “anxiety” or “arousal” or “empathy” or “fatigue” or “feeling” or “diazepam” or “egfr tki” or “imaging” or “epilepsy” or “ige-gtcs” or “response” or “addiction” or “alzheimer” or “anhedonia” or “apoptosis” or “awareness” or “cognition” or “cognitive” or “glutamate” or “hypnotics” or “imagining” or “memristor” or “mind-body” or “parkinson” or “anesthesia” or “fluoxetine” or “narcolepsy” or “psychiatry” or “alexithymia” or “cognitivism” or “daydreaming” or “impulsivity” or “mindfulness” or “mindreading” or “psychedelic” or “sensitivity” or “neuroscience” or “psychobiotic” or “psychometric” or “transcranial” or “ambidexterity” or “braak staging” or “consciousness” or “egfr mutation” or “hallucinogens” or “introspection” or “psychotherapy” or “somatosensory” or “anesthesiology” or “antidepressant” or “antipsychotics” or “focal seizures” or “guillain-barre” or “mind wandering” or “mind-wandering” or “self-reference” or “anaesthesiology” or “impulse control” or “neuroaesthetics” or “neuropsychology” or “neurostimulator” or “psychopathology” or “self-reflection” or “somatosensation” or “action-readiness” or “active inference” or “devic's syndrome” or “empathic concern” or “neurostimulation” or “apoptotic pathway” or “asperger syndrome” or “video eeg monitoring” or “craniocerebral trauma” or “excitation/inhibition” or “executive functioning” or “glasgow outcome scale” or “intelligence quotient” or “neurological disorder” or “perceptual decoupling” or “subcortical structure” or “central nervous system” or “cerebral hypoperfusion” or “communication disorder” or “disorders of the brain” or “dorsal premotor cortex” or “electroencephalography” or “endocannabinoid system” or “inhibitory interneuron” or “power spectra analysis” or “responsive stimulation” or “self-generated thought” or “acute confusional state” or “intellectual disability” or “medial forebrain bundle” or “perceptual organization” or “antipsychotic medication” or “brain cortical thickness” or “brain lymphatic drainage” or “brain storm optimization” or “brain-computer interface” or “brain-inspired computing” or “early start denver model” or “medial prefrontal cortex” or “adaptive resonance theory” or “psychosocial intervention” or “psychological construction” or “segmentation brain lesions” or “brain structural plasticity” or “cerebral amyloid angiopathy” or “cerebral embolic protection” or “cerebral glucose metabolism” or “functional connectivity mri” or “human cortical brain tissue” or “signed differential mapping” or “add-on antipsychotic therapy” or “catechol-o-methyltransferase” or “positron emission tomography” or “single neuron reconstruction” or “stereoelectroencephalography” or “thalamocortical connectivitymental illness” or “mental disorder” or “mental health” or “depression” or “schizophrenia” or “bipolar disorder” or “dementia” or “psychosis" |
