# Supplementary material for: Topics and trends in artificial intelligence assisted human brain research
Source: PLoS One. 2020 Apr 6;15(4):e0231192. doi: 10.1371/journal.pone.0231192 (PMC7135272; doi:10.1371/journal.pone.0231192)
Supplement: S3 Table — (DOCX) [file pone.0231192.s005.docx]

**S3 Table. Full names of the abbreviations (in capitals) in Table 4 in the main text.**

| **Abbreviations** | **Full names** |
| --- | --- |
| AD | Alzheimer’s Disease |
| ADHD | Attention-Deficit/Hyperactivity Disorder |
| ADNI | Alzheimer’s Disease Neuroimaging Initiative |
| ALFF | Amplitude of Low Frequency Fluctuation |
| AMCI | Amnestic Mild Cognitive Impairment |
| ANFIS | Adaptive Neural Fuzzy Inference System |
| ApEn | Approximate Entropy |
| ASD | Autism Spectrum Disorder |
| BBB | Blood Brain Barrier |
| BCI | Brain-Computer Interface |
| BD | Bipolar Disorder |
| BDNF | Brain-derived Neurotrophic Factor |
| BG | Basal Ganglia |
| BI | Bispectral Index |
| BMI | Brain Machine Interface |
| BVFTD | Behavioral Variant Frontotemporal Dementia |
| CAD | Computer-Aided Diagnosis |
| CJD | Creutzfeldt-Jakob Disease |
| CMB | Cerebral Micro-Bleed |
| CNN | Convolutional Neural Network |
| CSP | Common Spatial Pattern |
| CT | Computer Tomography |
| DBS | Deep Brain Stimulation |
| DCE-MRI | Dynamic Contrast Enhanced-Magnetic Resonance Imaging |
| DMRI | Diffusion Magnetic Resonance Imaging |
| DOA | Depth of Anesthesia |
| DTI | Diffusion Tensor Imaging |
| ELM | Extreme Learning Machine |
| EMD | Empirical Mode Decomposition |
| ERP | Event Related Potential |
| ERRP | Error-Related Potential |
| F-18-FET | brain Fluorine-18 Fluoro-Ethyl-Tyrosine |
| FALFF | Fractional Amplitude of Low Frequency Fluctuation |
| FC | Functional Connectivity |
| FCM | Fuzzy C-Means |
| FNIRS | Functional Near-Infrared Spectroscopy |
| FRN | Feedback-related Negativity |
| GBM | Glioblastoma Multiforme |
| GEPSVM | Generalized Eigenvalue Proximal Support Vector Machine |
| GLCM | Gray-Level Co-Occurrence Matrix |
| GM | Gray Matter |
| GMM | Gaussian Mixture Model |
| GTV | Gross Tumor Volume |
| GWAS | Genome-Wide Association Study |
| HARDI | High Angular Resolution Diffusion Imaging |
| HBO | Hemoglobin |
| HIE | Hypoxic Ischemic Encephalopathy |
| HIV | Human Immunodeficiency Virus |
| HTM | Hierarchical Temporal Memory |
| IBSR | Internet Brain Segmentation Repository |
| ICA | Independent Components Analysis |
| IED | Interictal Epileptiform Discharge |
| IEEG | Intracranial Electroencephalographic |
| IMF | Intrinsic Mode Function |
| IQ | Intelligence Quotient |
| MCI | Mild Cognitive Impairment |
| MCI-C | Mild Cognitive Impairment-Converter |
| MCI-NC | Mild Cognitive Impairment-Non-Conversion |
| MDD | Major Depressive Disorder |
| MI | Movement Imagery |
| MI-BCI | MotorImagery-Based Brain-Computer Interface |
| MMN | Mismatch Negativity |
| MRI-AC | Magnetic Resonance Image-based Attenuation Correction |
| MR-image | Magnetic Resonance image |
| MTLE | Mesial Temporal Lobe Epilepsy |
| MVPA | Multivoxel Pattern Analysis |
| NFT | Neurofeedback Training |
| NIRS | Near-Infrared Spectroscopy |
| NMF | Nonnegative Matrix Factorization |
| OCD | Obsessive-Compulsive Disorder |
| PD | Parkinson's Disease |
| PET | Positron Emission Tomography |
| PET/CT | Positron Emission Tomography/Computer Tomography |
| PET/MR | Positron Emission Tomography/Magnetic Resonance |
| PET/MRI | Positron Emission Tomography/Magnetic Resonance Imaging |
| PLV | Phase Locking Value |
| PNES | Psychogenic Non-Epileptic Seizures |
| PSO | Particle Swarm Optimization |
| PTSD | Posttraumatic Stress Disorder |
| PVS | Perivascular Space |
| RCBV | Relative Cerebral Blood Volume |
| ReHo | Regional Homogeneity |
| REM | Rapid Eye Movement |
| RHD | Right Hemisphere Brain Damage |
| RSN | Resting State Network |
| RSVP | Rapid Serial Visual Presentation |
| RTMS | Repetitive Transcranial Magnetic Stimulation |
| SEEG | Stereo Electroencephalography |
| SNN | Spiking Neural Networks |
| SOC | System-On-Chip |
| SSVEP | Steady-State Visually Evoked Potential |
| STDP | Spike Timing Dependent Plasticity |
| STN | Subthalamic Nucleus |
| STS | Superior Temporal Sulcus |
| SUV | Standardized Uptake Value |
| SVM | Support Vector Machine |
| SZ | Schizophrenia |
| TBI | Traumatic Brain Injury |
| TCD | Transcranial Doppler |
| TDCS | Transcranial Direct Current Stimulation |
| TDP-43 | Transactive response DNA-binding Protein of 43 kDa |
| TLE | Temporal Lobe Epilepsy |
| TLR | Toll Like Receptors |
