# Supplementary material for: Topics and trends in artificial intelligence assisted human brain research
Source: PLoS One. 2020 Apr 6;15(4):e0231192. doi: 10.1371/journal.pone.0231192 (PMC7135272; doi:10.1371/journal.pone.0231192)
Supplement: S4 Table — (DOCX) [file pone.0231192.s006.docx]

**S4 Table. Top countries/regions in the research field, ranked by paper count in a decreasing order.**

| **Name** | **AC** | **%** | **CC** | **H** | **>=300** | **>=100** | **>=50** | **2009-2013** | | **2014-2018** | |
| --- | --- | --- | --- | --- | --- | --- | --- | --- | --- | --- | --- |
|  |  |  |  |  |  |  |  | **AC (R)** | **CC** | **AC (R)** | **CC** |
| USA | 2,077 | 32.88 | 44,284 | 92 | 7 | 82 | 211 | 666 (1) | 7,886 | 1,411 (1) | 36,398 |
| China | 1,135 | 17.97 | 11,844 | 50 | 1 | 14 | 51 | 183 (4) | 1,096 | 952 (2) | 10,748 |
| UK | 702 | 11.11 | 15,354 | 62 | 3 | 29 | 80 | 237 (2) | 2,929 | 465 (3) | 12,425 |
| Germany | 575 | 9.10 | 10,803 | 52 | 0 | 18 | 58 | 188 (3) | 2,044 | 387 (4) | 8,759 |
| India | 391 | 6.19 | 3,858 | 29 | 0 | 4 | 16 | 61 (11) | 190 | 330 (5) | 3,668 |
| Canada | 377 | 5.97 | 5,864 | 36 | 0 | 10 | 26 | 107 (5) | 1,166 | 270 (6) | 4,698 |
| South Korea | 285 | 4.51 | 3,454 | 33 | 0 | 2 | 13 | 60 (12) | 267 | 225 (7) | 3,187 |
| Spain | 283 | 4.48 | 4,301 | 35 | 0 | 4 | 17 | 96 (7) | 731 | 187 (9) | 3,570 |
| France | 279 | 4.42 | 6,114 | 39 | 1 | 12 | 29 | 97 (6) | 1,324 | 182 (11) | 4,790 |
| Italy | 279 | 4.42 | 5,056 | 37 | 1 | 8 | 29 | 93 (9) | 862 | 186 (10) | 4,194 |
| Netherlands | 266 | 4.21 | 6,794 | 40 | 3 | 12 | 34 | 95 (8) | 1,384 | 171 (12) | 5,410 |
| Australia | 257 | 4.07 | 3,085 | 30 | 0 | 2 | 15 | 43 (15) | 285 | 214 (8) | 2,800 |
| Japan | 213 | 3.37 | 2,611 | 24 | 0 | 5 | 11 | 59 (13) | 437 | 154 (13) | 2,174 |
| Iran | 184 | 2.91 | 2,214 | 25 | 0 | 4 | 10 | 64 (10) | 372 | 120 (15) | 1,842 |
| Switzerland | 170 | 2.69 | 3,224 | 30 | 0 | 6 | 20 | 46 (14) | 506 | 124 (14) | 2,718 |
| Taiwan | 126 | 1.99 | 1,555 | 20 | 0 | 2 | 5 | 40 (17) | 265 | 86 (17) | 1,290 |
| Singapore | 120 | 1.90 | 2,953 | 31 | 0 | 5 | 19 | 43 (16) | 517 | 77 (20) | 2,436 |
| Turkey | 118 | 1.87 | 1,728 | 20 | 0 | 3 | 7 | 37 (18) | 277 | 81 (18) | 1,451 |
| Belgium | 108 | 1.71 | 1,358 | 22 | 0 | 0 | 4 | 30 (21) | 181 | 78 (19) | 1,177 |
| Malaysia | 105 | 1.66 | 1,059 | 18 | 0 | 1 | 2 | 17 (27) | 58 | 88 (16) | 1,001 |

**Abbreviations:** R: ranking position; AC: paper count; %: percentage of papers in the research field; CC: citation count; H: H index; >=300, >=100, >=50: counts of papers with more than 300, 100, or 50 citations.
