# Supplementary material for: Topics and trends in artificial intelligence assisted human brain research
Source: PLoS One. 2020 Apr 6;15(4):e0231192. doi: 10.1371/journal.pone.0231192 (PMC7135272; doi:10.1371/journal.pone.0231192)
Supplement: S5 Table — Numbers outside brackets are measure values, those within brackets are ranks of countries/regions by the corresponding measure values in a decreasing order. (DOCX) [file pone.0231192.s007.docx]

**S5 Table. Values of centrality measures for the 20 most influential countries/regions in the collaboration network.** Note: Numbers outside brackets are measure values, those within brackets are ranks of countries/regions by the corresponding measure values in a decreasing order.

| **Country/Region** | **Degree** | **Closeness** | **Betweenness** | **Eigencentrality** |
| --- | --- | --- | --- | --- |
| USA | 1258 (1) | 0.053 (1) | 0.06612 (1) | 0.6031 (1) |
| UK | 650 (2) | 0.053 (1) | 0.01625 (2) | 0.3418 (3) |
| China | 583 (3) | 0.053 (1) | 0.00833 (3) | 0.2894 (4) |
| Germany | 515 (4) | 0.053 (1) | 0.00297 (4) | 0.2307 (5) |
| Canada | 359 (5) | 0.053 (1) | 0.00266 (7) | 0.1567 (8) |
| France | 309 (6) | 0.053 (1) | 0.00290 (5) | 0.1661 (7) |
| Netherlands | 269 (7) | 0.053 (1) | 0.00151 (10) | 0.0675 (14) |
| Italy | 264 (8) | 0.050 (8) | 0.00232 (8) | 0.4484 (2) |
| Australia | 238 (9) | 0.050 (8) | 0.00277 (6) | 0.1475 (10) |
| Spain | 209 (10) | 0.050 (8) | 0.00041 (15) | 0.0562 (17) |
| South Korea | 204 (11) | 0.048 (11) | 0.00127 (11) | 0.1191 (11) |
| Switzerland | 182 (12) | 0.048 (11) | 0.00042 (14) | 0.0637 (15) |
| Singapore | 139 (13) | 0.048 (11) | 0.00039 (16) | 0.0592 (16) |
| Belgium | 127 (14) | 0.045 (14) | 0.00047 (13) | 0.1129 (12) |
| Japan | 109 (15) | 0.045 (14) | 0.00051 (12) | 0.1774 (6) |
| India | 107 (16) | 0.045 (14) | 0.00016 (17) | 0.0744 (13) |
| Denmark | 105 (17) | 0.043 (17) | 0.00152 (9) | 0.1497 (9) |
| Austria | 87 (18) | 0.042 (18) | 0.00010 (18) | 0.0462 (19) |
| Iran | 69 (19) | 0.042 (18) | 0.00004 (19) | 0.0274 (20) |
| Greece | 51 (20) | 0.040 (20) | 0.00000 (20) | 0.0556 (18) |
